# Supplementary material for: Electronic Health Records, Interoperability and Patient Safety in Health Systems of High-income Countries: A Systematic Review Protocol
Source: BMJ Open. 2021 Jul 14;11(7):e044941. doi: 10.1136/bmjopen-2020-044941 (PMC8280868; doi:10.1136/bmjopen-2020-044941)
Supplement: Supplementary data [file bmjopen-2020-044941supp001.pdf]

Supplement 1: Search strategy exploring main themes, utilizing search terms and related terminology derivations for each theme (electronic health records, interoperability, and patient safety).

#	Searches
1	exp Medical Records Systems, Computerized/ or exp Electronic Health Records/ or exp Hospital Information Systems/
2	limit 1 to (english language and yr="2010 - 2020")
3	exp Electronic Health Records/
4	limit 3 to (english language and yr="2010 - 2020")
5	exp Health Information Exchange/ or exp Medical Informatics/ or exp Decision Support Systems, Clinical/ or exp Medical Records Systems, Computerized/ or exp Medical Record Linkage/
6	limit 5 to (english language and yr="2010 - 2020")
7	exp Medical Informatics/
8	limit 7 to (english language and yr="2010 - 2020")
9	exp Hospital Information Systems/
10	limit 9 to (english language and yr="2010 - 2020")
11	exp Medical Informatics/
12	limit 11 to (english language and yr="2010 - 2020")
13	exp Health Information Interoperability/
14	limit 13 to (english language and yr="2010 - 2020")
15	exp Systems Integration/
16	limit 15 to (english language and yr="2010 - 2020")
17	exp Patient Safety/
18	limit 17 to (english language and yr="2010 - 2020")
19	(Patient adj1 incident*).mp. [mp=title, abstract, original title, name of substance word, subject heading word, floating sub-heading word, keyword heading word, organism supplementary concept word, protocol supplementary concept word, rare disease supplementary concept word, unique identifier, synonyms]
20	limit 19 to (english language and yr="2010 - 2020")
21	(Adverse adj1 event*).mp. [mp=title, abstract, original title, name of substance word, subject heading word, floating sub-heading word, keyword heading word, organism supplementary

	concept word, protocol supplementary concept word, rare disease supplementary concept word, unique identifier, synonyms]
22	limit 21 to (english language and yr="2010 - 2020")
23	(Patient adj1 outcome*).mp. [mp=title, abstract, original title, name of substance word, subject heading word, floating sub-heading word, keyword heading word, organism supplementary concept word, protocol supplementary concept word, rare disease supplementary concept word, unique identifier, synonyms]
24	limit 23 to (english language and yr="2010 - 2020")
25	(Patient adj1 harm).mp. [mp=title, abstract, original title, name of substance word, subject heading word, floating sub-heading word, keyword heading word, organism supplementary concept word, protocol supplementary concept word, rare disease supplementary concept word, unique identifier, synonyms]
26	limit 25 to (english language and yr="2010 - 2020")
27	exp Risk Management/
28	limit 27 to (english language and yr="2010 - 2020")
29	2 or 4 or 6 or 8 or 10 or 12
30	14 or 16
31	18 or 20 or 22 or 24 or 26 or 28
32	29 and 30 and 31
