# Supplementary material for: Electronic Health Records, Interoperability and Patient Safety in Health Systems of High-income Countries: A Systematic Review Protocol
Source: BMJ Open. 2021 Jul 14;11(7):e044941. doi: 10.1136/bmjopen-2020-044941 (PMC8280868; doi:10.1136/bmjopen-2020-044941)
Supplement: Author's manuscript [file bmjopen-2020-044941.draft_revisions.pdf]

BMJ Open

BMJ Open is committed to open peer review. As part of this commitment we make the peer review history of every article we publish publicly available.

When an article is published we post the peer reviewers' comments and the authors' responses online. We also post the versions of the paper that were used during peer review. These are the versions that the peer review comments apply to.

The versions of the paper that follow are the versions that were submitted during the peer review process. They are not the versions of record or the final published versions. They should not be cited or distributed as the published version of this manuscript.

BMJ Open is an open access journal and the full, final, typeset and author-corrected version of record of the manuscript is available on our site with no access controls, subscription charges or pay-per-view fees (http://bmjopen.bmj.com).

If you have any questions on BMJ Open's open peer review process please email info.bmjopen@bmj.com

BMJ Open

Electronic Health Records, Interoperability, and Patient Safety in Health Systems of High-Income Countries: A Systematic Review Protocol

Journal:	*BMJ Open*
Manuscript ID	bmjopen-2020-044941
Article Type:	Protocol
Date Submitted by the Author:	19-Sep-2020
Complete List of Authors:	Li, Edmond; Imperial College London, Patient Safety Translational Research Centre, Institute of Global Health Innovation, Department of Surgery & Cancer; London School of Hygiene and Tropical Medicine Faculty of Infectious and Tropical Diseases, Clinical Research Department<br>Neves, Ana Luisa; Imperial College London, Patient Safety Translational Research Centre, Institute of Global Health Innovation, Department of Surgery & Cancer; University of Porto, Center for Health Technology and Services Research / Department of Community Medicine, Health Information and Decision<br>Clarke, Jonathan; Imperial College London, Patient Safety Translational Research Centre, Institute of Global Health Innovation, Department of Surgery & Cancer; Imperial College London, Centre for Mathematics of Precision Healthcare<br>Ashrafian, Hutan; Imperial College London, Patient Safety Translational Research Centre, Institute of Global Health Innovation, Department of Surgery & Cancer<br>Darzi, Ara; Imperial College London, Patient Safety Translational Research Centre, Institute of Global Health Innovation, Department of Surgery & Cancer
Keywords:	Health informatics < BIOTECHNOLOGY & BIOINFORMATICS, Telemedicine < BIOTECHNOLOGY & BIOINFORMATICS, Information technology < BIOTECHNOLOGY & BIOINFORMATICS, Information management < BIOTECHNOLOGY & BIOINFORMATICS, HEALTH SERVICES ADMINISTRATION & MANAGEMENT, Health policy < HEALTH SERVICES ADMINISTRATION & MANAGEMENT

SCHOLARONE™
Manuscripts

I, the Submitting Author has the right to grant and does grant on behalf of all authors of the Work (as defined in the below author licence), an exclusive licence and/or a non-exclusive licence for contributions from authors who are: i) UK Crown employees; ii) where BMJ has agreed a CC-BY licence shall apply, and/or iii) in accordance with the terms applicable for US Federal Government officers or employees acting as part of their official duties; on a worldwide, perpetual, irrevocable, royalty-free basis to BMJ Publishing Group Ltd ("BMJ") its licensees and where the relevant Journal is co-owned by BMJ to the co-owners of the Journal, to publish the Work in this journal and any other BMJ products and to exploit all rights, as set out in our licence.

The Submitting Author accepts and understands that any supply made under these terms is made by BMJ to the Submitting Author unless you are acting as an employee on behalf of your employer or a postgraduate student of an affiliated institution which is paying any applicable article publishing charge ("APC") for Open Access articles. Where the Submitting Author wishes to make the Work available on an Open Access basis (and intends to pay the relevant APC), the terms of reuse of such Open Access shall be governed by a Creative Commons licence – details of these licences and which Creative Commons licence will apply to this Work are set out in our licence referred to above.

Other than as permitted in any relevant BMJ Author's Self Archiving Policies, I confirm this Work has not been accepted for publication elsewhere, is not being considered for publication elsewhere and does not duplicate material already published. I confirm all authors consent to publication of this Work and authorise the granting of this licence.

For peer review only

Electronic Health Records, Interoperability, and Patient Safety in Health Systems of High-Income Countries: A Systematic Review Protocol

Edmond Li[1], Ana Luisa Neves[1,2], Jonathan Clarke[1,3], Hutan Ashrafian[1], Ara Darzi[1]

[1] Imperial NIHR Patient Safety Translational Research Centre (PSTRC), Institute of Global Health Innovation (IGHI), Imperial College London, London, United Kingdom

[2] Department of Community Medicine, Information and Health Decision Sciences (MEDCIDS) / Center for Health Technology and Services Research (CINTESIS), University of Porto, Porto, Portugal.

[3] Centre for Mathematics of Precision Healthcare, Imperial College London, London, United Kingdom

For peer review only

Corresponding author

Name: Edmond Li

Email: edmond.li19@imperial.ac.uk

Address: Institute of Global Health Innovation, Room 1035/7, QEQM Wing, St Mary's Campus, London W2 1NY

Keywords [MeSH terms]: electronic health records, electronic medical records, computerised patient records, health information technology, health information exchanges, interoperability, patient safety, patient harm, adverse events, health outcomes

Word count: 1821

Target journal: BMJ Open

Abstract

Introduction

The availability and routine use of electronic health records (EHRs) have become commonplace in healthcare systems of many high-income countries. While there is an ever-growing body of literature pertaining to EHR use, evidence surrounding the importance of EHR interoperability and its impact on patient safety remains less clear. There is therefore a need and opportunity to evaluate the evidence available regarding this relationship so as to better inform health informatics development and policies in the years to come.

Objective

This systematic review aims to evaluate the impact of EHR interoperability on patient safety in health systems of high-income countries.

Methods and analysis

A systematic literature review will be conducted via a computerised search through three databases: PubMed, Embase, and HMIC for relevant articles published between March 2010 and March 2020. Outcomes of interest will include: impact on patient safety, and the broader effects on health systems. Quality of the randomised quantitative studies will be assessed using Cochrane Risk of Bias Tool. Non-randomised papers will be evaluated with the Risk of Bias In Non-Randomised Studies - of Interventions (ROBINS-I) tool. Drummond's Checklist will be utilised for publications pertaining to economic evaluation. The National Institute for Health and Care Excellence (NICE) quality appraisal checklist will be used to assess qualitative studies. A narrative synthesis will be conducted for included studies, and the body of evidence will be summarised in a summary of findings table.

Ethics and dissemination

This review will summarise published studies with non-identifiable data and therefore does not require ethical approval. This protocol complies with the Preferred Reporting Items for Systematic Review and Meta-Analyses Protocols guidelines. Findings will be disseminated through preprints, open access peer-reviewed publication, and conference presentations. Patients or members of the public were not involved in the design of this study.

Strengths and limitations of this study

**Strengths**	**Limitations**
• Comprehensive characterisation of interventions using interoperable EHRs<br>• Summary and quality appraisal of existing evidence on the potential impact on patient safety<br>• Inclusion of both quantitative and qualitative methods can provide a comprehensive overview of the multitude of ways in which interoperable EHRs may affect patient safety and health systems.	• The heterogeneity of methods and outcomes assessed may obscure the true effect interoperable EHRs have had on patient safety in the clinical setting<br>• Potential small sample size in subgroup analyses, may negatively impact the statistical power in quantitative data synthesis.

For peer review only

For peer review only - http://bmjopen.bmj.com/site/about/guidelines.xhtml

• The proposed review attempts to answer a pragmatic question which is integral to influencing future health informatics development and policies.	

PROSPERO registration number [CRD42020209285]

This is an open access article distributed in accordance with the Creative Commons Attribution 4.0 Unported (CC BY 4.0) license, which permits others to copy, redistribute, remix, transform and build upon this work for any purpose, provided the original work is properly cited, a link to the licence is given, and indication of whether changes were made. See: https://creativecommons.org/licenses/by/4.0/.

For peer review only

For peer review only - http://bmjopen.bmj.com/site/about/guidelines.xhtml

Introduction

Electronic health records (EHRs) have become an integral part of modern healthcare since their initial mainstream implementation in the mid-late 2000s through the passing of the Health Information Technology for Economic and Clinical Health Act (HITECH) in the United States and the NHS National Programme for IT initiative (NPfIT) in England (1–4). From the documentation & retrieval of patient records and the prescription of medications, to coordinating complex care plans between different healthcare providers and electronic billing, EHRs fulfil a multitude of roles for both clinicians and patients alike (5–9).

In order to achieve EHR's full potential, it is critical to improve interoperability - i.e. "*the ability of health information systems to work together within and across organisation boundaries in order to advance effective delivery of healthcare for individuals and communities*" (10). The lack of universal interoperability is often cited as one of the many significant shortcomings of EHRs currently in use, resulting in duplication in healthcare costs, increased clinician workload fatigue, and poses a potential risk to patient safety (2). This is especially problematic for patient populations with chronic conditions, polypharmacy, and multiple comorbidities who are reliant upon effective patient information sharing via EHRs to facilitate their care (11).

In the fragmented EHR landscape of the United Kingdom, understanding this poor interoperability and accurately measuring its cost both to patient safety and the health system as a whole, remain challenging (12). Although there is a growing body of literature investigating areas such as the facilitators and barriers to EHR greater adoption, technical capabilities, and usability (13,14), no systematic review has been conducted exploring specifically the problem of interoperability amongst the assortment of EHRs in use, how it affects patient safety, and ultimately the financial cost savings lost to health systems.

In a recent systematic review by Dobrow *et al*. assessing the effects of EHR and HIT interoperability on health systems, 130 publications were included, with the majority being studies conducted in the United States, utilised quantitative methods, and focussed primarily in acute healthcare settings. The authors noted the use of interoperable EHRs had a positive impact on outcomes measures such as quality of care and productivity (13). However, in domains such as stakeholder engagement, performance & reliability, security & privacy, information quality, and ease of use, the benefits of interoperable EHRs was less clear (13). Amongst the 130 publications, 17 were reviews with the majority directed at exploring facilitators & barriers to EHR implementation, and the general benefits and impact of EHR use. While this review did focus on studies pertaining to the topic of interoperable EHRs, this was done so only from a broad perspective and included studies exploring a wide range of outcomes related to the effects of EHR on healthcare rather than specifically on their implications to patient safety.

In another review by Hersh *et al.*, the authors explored how health information exchange (HIE) affected health systems on a variety of domains, including costs, healthcare utilisation, health outcomes, healthcare worker attitudes, and sustainability. Despite the widespread routinely use of HIE, the authors described a general lack of robust evidence on the quality, costs, efficiency, usage, and sustainability (15). However, there was some evidence demonstrating HIEs being

For peer review only - http://bmjopen.bmj.com/site/about/guidelines.xhtml

associated with reduced utilisation and costs in emergency care settings despite methodological issues being present in many of the included publications (15). Although this review was ambitious in the wide scope of interest regarding the effects of HIE use, patient safety was not a primary topic of focus. Another limitation of this study was that it only contained US-based publications, and thus findings lack generalisability internationally to other health systems in high-income countries (HIC) which are both organised, financed differently.

Research aim

The overall aim of this literature review is to explore how EHR interoperability impacts patient safety, in the context of health systems in HICs. The results generated will aim to inform healthcare policymakers and help shape more effective EHR system implementation and modernisation efforts in the coming years.

Methods and analysis

Search strategy

A computerised search of the literature published in the last 10 years (March 2010 to March 2020) search will be performed on PubMed, Embase and Health Management Information Consortium (HMIC). The list of search strings used will include both free text and controlled terms, whenever supported (Table 1) and will be iteratively refined in consultation with the Imperial College St. Mary's campus medical librarian.

Grey literature sources will also be searched, including registrations in the International Prospective Register of Systematic Reviews, reports of relevant stakeholder organisations (NHS England, American Medical Informatics Association (AMIA), eHealth at WHO, and conference proceedings (last 5 years) of several related conferences (AMIA, MedInfo, Medicine 2.0, Medicine X)), in order to identify possible additional studies that meet the inclusion criteria.

The search has also been restricted to HIC and articles published in English only.

Table 1: Concepts and database search terms

**Electronic health records**		**Interoperability**		**Patient safety**
• Electronic health record* / EHR<br>• Electronic medical record* / EMR<br>• Health information exchange / HIE<br>• Health information technology / HIT<br>• EHR-based interventions	**AND**	• Interoperabl*<br>• Interoperability<br>• Standards of information	**AND**	• Patient safety<br>• Patient adj1 incident*<br>• Adverse adj1 event*<br>• Patient adj1 outcome*<br>• Patient adj1 harm<br>• Risk management

For peer review only

• Computerised patient record* / CPR				

Study selection criteria

A summary of the population, intervention, comparison, outcomes, and type of studies being considered is provided in **Table 2**. This systematic review will focus on studies performed in high-income countries and published in English only. Studies assessing the impact of EHR interoperability will be included. Interventions will include EHR systems interoperable with other health information technology systems both within and across healthcare facilities, as well as those used in tertiary and community settings. The primary outcomes to be considered in this review will be safety outcomes, including adverse events/incidents, safety-related patient experiences, and health outcomes. In addition, secondary outcomes would include studies exploring the broader impact of interoperable EHRs on health systems such as cost effectiveness and clinical culture amongst healthcare providers on the topic. Both qualitative and quantitative studies will be included. Reference lists of the selected articles will also be screened for papers which may have been missed by the initial database search but still meet the eligibility criteria.

Table 2: PICO inclusion criteria

Population	High-income countries utilising electronic health records
Intervention	EHRs with interoperability
Comparison	Usual care (i.e. existing baseline of interoperability)
Outcome	Impact on patient safety and quality of care

Screening

Articles to be included will be screened by two independent reviewers, following the process described in the Preferred Reporting Items for Systematic Reviews and Meta-Analyses (PRISMA) flow diagram (16). The initial screening will be done by the first reviewer based on the publication titles, followed by a second screening based on the abstracts. Included abstracts will then be fully reviewed by two independent researchers to produce a unified selection of articles to be included in this review. Cohen's kappa will be calculated to ensure inter-rater agreement and consistency in the selection of studies to be included (17,18). Any disagreements will be resolved by consensus; if a Cohen's kappa value of less than 0.6 is reported, the discrepancies will be addressed through discussions with a more experienced third investigator.

Data extraction & analysis

Data extraction will be performed using a standardised extraction table for each of the two investigators to summarise the characteristics and findings of each included study, including name of the first author, year of publication, study design, number of participants, retention rates, setting characteristics, outcome measures, and main results. The content of the two summary tables will then be aggregated and reviewed once more by both investigators, with any disagreements being solved by the third senior investigator.

Quality assessment

The quality of randomised controlled trials and cluster randomised trials will be assessed using the Cochrane Risk of Bias Tool (19), and the quality of non-randomised intervention studies (i.e., case control, cohort, quasi-experimental) will be appraised using the 'Risk of Bias In Non-

For peer review only - http://bmjopen.bmj.com/site/about/guidelines.xhtml

Randomised Studies - of Interventions' (ROBINS-I) tool (20). For cost-effectiveness studies, the Drummond's checklist for assessing economic evaluations will be used (21). The National Institute for Health and Care Excellence (NICE) quality appraisal checklist will be utilised to assess the selected qualitative studies (22). Two independent reviewers will score the selected studies and any disagreements will be resolved by a third person. A risk of bias table along with an overall, collective bias narrative will be produced to summarise the biases of outcomes observed amongst the evaluated studies.

Descriptive analysis

A narrative synthesis will be conducted for all the included studies.
Quantitative studies with comparable outcome measures will be aggregated to allow for comparative analyses whenever possible. However, no meta-analysis will be done for this systematic review as the likely heterogeneity of the outcomes would make this impractical. Qualitative studies will be thematically analysed. Those that demonstrate related, prominent themes in their findings will be grouped together and collectively summarised via a thematic narrative. The body of evidence will be summarised in two separate Summary of Findings tables (for both qualitative and quantitative studies) in accordance to the 'Grading of Recommendations Assessment, Development and Evaluation' (GRADE) criteria where possible (23).

Patient and public involvement

This systematic literature review saw no direct participation by patients or the public during the design of this study. However, this study was designed following a series of structured interviews with patients regarding their experience of attending multiple institutions for hospital care (24). As this literature review will be used to form the basis for subsequent studies exploring the topic including ones involving patients, findings from this review will be shared with patient research groups to gain feedback and encourage further discourse surrounding the topic of EHR interoperability and patient safety.

Amendments

Any amendments to this protocol will be documented with reference to saved searches and analysis methods, which will be recorded in bibliographic databases, Mendeley and Excel templates for data collection and synthesis.

Discussion

One of the primary strengths stemming from the almost exploratory nature of this systematic review is the ability to generate a succinct, comprehensive appraisal of the best evidence currently available regarding how EHR interoperability impacts patient care and safety. By publishing this review protocol beforehand, we demonstrate a clear, robust, and transparent approach to aggregating the anticipated assortment of literature on the subject in question.
There are also some potential limitations to be acknowledged. A potential challenge would be the likely anticipated heterogeneity in methodology of the included articles. With such diverse means of measuring and assessing the effects of EHR interoperability, this will likely make comparisons between studies difficult and may obscure the true measure of effect EHR interoperability has had in the clinical setting. To mitigate this risk, outcomes will be grouped whenever possible, and summarised as a narrative synthesis. However, this can also represent a strength as it will provide a comprehensive overview on the subject, capitalising on various research methodologies, and providing novel insights on the impact of interoperable EHR systems on patient safety.

For peer review only - http://bmjopen.bmj.com/site/about/guidelines.xhtml

Acknowledgments

We would like to thank Michael Gainsford (Library Manager and Liaison Librarian at Imperial College London) for his support and guidance provided to improve the composition of the search terms and procedural aspects of the overall search strategy. JC acknowledges support from the Wellcome Trust [215938/Z/19/Z].

Funding

This research was supported through the Imperial College National Institute for Health Research (NIHR) Patient Safety Translational Research Centre (PSTRC) and the Imperial College Biomedical Research Centre (BRC). However, the funder/sponsor has had no role in development and drafting of this protocol.

For peer review only

For peer review only - http://bmjopen.bmj.com/site/about/guidelines.xhtml

References

1. Colicchio TK, Cimino JJ, Del Fiol G. Unintended consequences of nationwide electronic health record adoption: Challenges and opportunities in the post-meaningful use era. *Journal of Medical Internet Research*. [Online] 2019;21(6): 1–9. Available from: doi:10.2196/13313
2. Roman LC, Ancker JS, Johnson SB, Senathirajah Y. Navigation in the electronic health record: A review of the safety and usability literature. *Journal of Biomedical Informatics*. [Online] Elsevier Inc.; 2017;67: 69–79. Available from: doi:10.1016/j.jbi.2017.01.005
3. Justinia T. The UK's National Programme for IT: Why was it dismantled? *Health Services Management Research*. [Online] 2017;30(1): 2–9. Available from: doi:10.1177/0951484816662492
4. Wachter RM. Making IT work : harnessing the power of health IT to improve care in England. *National Advisory Group on Health Information Technology in England*. [Online] 2016; 71. Available from: https://www.gov.uk/government/uploads/system/uploads/attachment_data/file/550866/Wachter_Review_Accessible.pdf%0Ahttps://assets.publishing.service.gov.uk/government/uploads/system/uploads/attachment_data/file/550866/Wachter_Review_Accessible.pdf
5. Kruse CS, Kristof C, Jones B, Mitchell E, Martinez A. Barriers to Electronic Health Record Adoption: a Systematic Literature Review. *Journal of Medical Systems*. [Online] Journal of Medical Systems; 2016;40(12). Available from: doi:10.1007/s10916-016-0628-9
6. Ayaad O, Alloubani A, ALhajaa EA, Farhan M, Abuseif S, Al Hroub A, et al. The role of electronic medical records in improving the quality of health care services: Comparative study. *International Journal of Medical Informatics*. [Online] Elsevier; 2019;127(April): 63–67. Available from: doi:10.1016/j.ijmedinf.2019.04.014
7. Evans RS. Electronic Health Records: Then, Now, and in the Future. *Yearbook of Medical Informatics*. [Online] 2016;25(S 01): S48–S61. Available from: doi:10.15265/IYS-2016-s006
8. Gagnon MP, Payne-Gagnon J, Breton E, Fortin JP, Khoury L, Dolovich L, et al. Adoption of electronic personal health records in Canada: Perceptions of stakeholders. *International Journal of Health Policy and Management*. [Online] 2016;5(7): 425–433. Available from: doi:10.15171/ijhpm.2016.36
9. Campanella P, Lovato E, Marone C, Fallacara L, Mancuso A, Ricciardi W, et al. The impact of electronic health records on healthcare quality: a systematic review and meta-analysis. *The European Journal of Public Health*. [Online] 2016;26(1): 60–64. Available from: doi:10.1093/eurpub/ckv122
10. Reis ZSN, Maia TA, Marcolino MS, Becerra-Posada F, Novillo-Ortiz D, Ribeiro ALP. Is There Evidence of Cost Benefits of Electronic Medical Records, Standards, or Interoperability in Hospital Information Systems? Overview of Systematic Reviews. *JMIR Medical Informatics*. [Online] 2017;5(3): e26. Available from: doi:10.2196/medinform.7400
11. World Health Organization (WHO). *eHealth in the Region of the Americas: breaking down the barriers to implementation*. [Online] 2016. Available from: http://iris.paho.org/xmlui/bitstream/handle/123456789/31286/9789275119259-eng.pdf?sequence=6&isAllowed=y
12. McAlearney AS, Sieck C, Hefner J, Robbins J, Huerta TR. Facilitating Ambulatory

For peer review only - http://bmjopen.bmj.com/site/about/guidelines.xhtml

Electronic Health Record System Implementation: Evidence from a Qualitative Study. *BioMed Research International*. [Online] 2013;2013: 1–9. Available from: doi:10.1155/2013/629574
13. Dobrow MJ, Bytautas JP, Tharmalingam S, Hagens S. Interoperable Electronic Health Records and Health Information Exchanges: Systematic Review. *JMIR Medical Informatics*. [Online] 2019;7(2): e12607. Available from: doi:10.2196/12607
14. Zahabi M, Kaber DB, Swangnetr M. Usability and Safety in Electronic Medical Records Interface Design: A Review of Recent Literature and Guideline Formulation. *Human Factors*. [Online] 2015;57(5): 805–834. Available from: doi:10.1177/0018720815576827
15. Hersh WR, Totten AM, Eden KB, Devine B, Gorman P, Kassakian SZ, et al. Outcomes From Health Information Exchange: Systematic Review and Future Research Needs. *JMIR Medical Informatics*. [Online] 2015;3(4): e39. Available from: doi:10.2196/medinform.5215
16. Moher D, Liberati A, Tetzlaff J, Altman DG, Altman D, Antes G, et al. Preferred reporting items for systematic reviews and meta-analyses: The PRISMA statement. *PLoS Medicine*. [Online] 2009;6(7). Available from: doi:10.1371/journal.pmed.1000097
17. Landis JR, Koch GG. The Measurement of Observer Agreement for Categorical Data. *Biometrics*. [Online] 1977;33(1): 159. Available from: doi:10.2307/2529310
18. McHugh ML. Interrater reliability: the kappa statistic. *Biochemia medica*. 2012;22(3): 276–282.
19. Sterne JAC, Savović J, Page MJ, Elbers RG, Blencowe NS, Boutron I, et al. RoB 2: A revised tool for assessing risk of bias in randomised trials. *The BMJ*. [Online] 2019;366(August). Available from: doi:10.1136/bmj.l4898
20. Sterne JA, Hernán MA, Reeves BC, Savović J, Berkman ND, Viswanathan M, et al. ROBINS-I: A tool for assessing risk of bias in non-randomised studies of interventions. *BMJ (Online)*. [Online] 2016;355: 4–10. Available from: doi:10.1136/bmj.i4919
21. Drummond MF, Brien BJ, Torrance GW, Stoddart GL. *Methods for the Economic Evaluation of Health Care Programmes*.. 2nd ed. Oxford University Press; 1997.
22. *Methods for the development of NICE public health guidance*. [Online]. 3rd n. National Institute for Clinical Excellence (NICE); 2018. Available from: https://www.nice.org.uk/process/pmg4/resources/methods-for-the-development-of-nice-public-health-guidance-third-edition-pdf-2007967445701
23. Guyatt GH, Oxman AD, Vist GE, Kunz R, Falck-Ytter Y, Alonso-Coello P, et al. GRADE: an emerging consensus on rating quality of evidence and strength of recommendations. *BMJ*. [Online] 2008;336(7650): 924–926. Available from: doi:10.1136/bmj.39489.470347.AD
24. Warren LR, Harrison M, Arora S, Darzi A. Working with patients and the public to design an electronic health record interface: A qualitative mixed-methods study. *BMC Medical Informatics and Decision Making*. [Online] BMC Medical Informatics and Decision Making; 2019;19(1): 1–8. Available from: doi:10.1186/s12911-019-0993-7

For peer review only - http://bmjopen.bmj.com/site/about/guidelines.xhtml

PRISMA-P (Preferred Reporting Items for Systematic review and Meta-Analysis Protocols) 2015 checklist: recommended items to address in a systematic review protocol*

Section and topic	Item No.	Checklist item	Reported on Page No.
**ADMINISTRATIVE INFORMATION**			
Title:			
Identification	1a	Identify the report as a protocol of a systematic review	1
Update	1b	If the protocol is for an update of a previous systematic review, identify as such	N/A
Registration	2	If registered, provide the name of the registry (such as PROSPERO) and registration number	3
Authors:			
Contact	3a	Provide name, institutional affiliation, e-mail address of all protocol authors; provide physical mailing address of corresponding author	1
Contributions	3b	Describe contributions of protocol authors and identify the guarantor of the review	1
Amendments	4	If the protocol represents an amendment of a previously completed or published protocol, identify as such and list changes; otherwise, state plan for documenting important protocol amendments	N/A
Support:			
Sources	5a	Indicate sources of financial or other support for the review	8
Sponsor	5b	Provide name for the review funder and/or sponsor	8
Role of sponsor or funder	5c	Describe roles of funder(s), sponsor(s), and/or institution(s), if any, in developing the protocol	8
**INTRODUCTION**			
Rationale	6	Describe the rationale for the review in the context of what is already known	4-5
Objectives	7	Provide an explicit statement of the question(s) the review will address with reference to participants, interventions, comparators, and outcomes (PICO)	5
**METHODS**			
Eligibility criteria	8	Specify the study characteristics (such as PICO, study design, setting, time frame) and report characteristics (such as years considered, language, publication status) to be used as criteria for eligibility for the review	5-6
Information sources	9	Describe all intended information sources (such as electronic databases, contact with study authors, trial registers or other grey literature sources) with planned dates of coverage	5
Search strategy	10	Present draft of search strategy to be used for at least one electronic database, including planned limits, such that it could be repeated	5

For peer review only

Study records:			
Data management	11a	Describe the mechanism(s) that will be used to manage records and data throughout the review	6-7
Selection process	11b	State the process that will be used for selecting studies (such as two independent reviewers) through each phase of the review (that is, screening, eligibility and inclusion in meta-analysis)	6
Data collection process	11c	Describe planned method of extracting data from reports (such as piloting forms, done independently, in duplicate), any processes for obtaining and confirming data from investigators	6-7
Data items	12	List and define all variables for which data will be sought (such as PICO items, funding sources), any pre-planned data assumptions and simplifications	6
Outcomes and prioritization	13	List and define all outcomes for which data will be sought, including prioritization of main and additional outcomes, with rationale	6
Risk of bias in individual studies	14	Describe anticipated methods for assessing risk of bias of individual studies, including whether this will be done at the outcome or study level, or both; state how this information will be used in data synthesis	6-7
Data synthesis	15a	Describe criteria under which study data will be quantitatively synthesised	6-7
	15b	If data are appropriate for quantitative synthesis, describe planned summary measures, methods of handling data and methods of combining data from studies, including any planned exploration of consistency (such as I^2, Kendall's τ)	7
	15c	Describe any proposed additional analyses (such as sensitivity or subgroup analyses, meta-regression)	-
	15d	If quantitative synthesis is not appropriate, describe the type of summary planned	7
Meta-bias(es)	16	Specify any planned assessment of meta-bias(es) (such as publication bias across studies, selective reporting within studies)	6-7
Confidence in cumulative evidence	17	Describe how the strength of the body of evidence will be assessed (such as GRADE)	7

*** It is strongly recommended that this checklist be read in conjunction with the PRISMA-P Explanation and Elaboration (cite when available) for important clarification on the items. Amendments to a review protocol should be tracked and dated. The copyright for PRISMA-P (including checklist) is held by the PRISMA-P Group and is distributed under a Creative Commons Attribution Licence 4.0.**

From: Shamseer L, Moher D, Clarke M, Ghersi D, Liberati A, Petticrew M, Shekelle P, Stewart L, PRISMA-P Group. Preferred reporting items for systematic review and meta-analysis protocols (PRISMA-P) 2015: elaboration and explanation. BMJ. 2015 Jan 2;349(jan02 1):g7647.

For peer review only

BMJ Open

Electronic Health Records, Interoperability, and Patient Safety in Health Systems of High-Income Countries: A Systematic Review Protocol

Journal:	*BMJ Open*
Manuscript ID	bmjopen-2020-044941.R1
Article Type:	Protocol
Date Submitted by the Author:	04-Feb-2021
Complete List of Authors:	Li, Edmond; Imperial College London, Patient Safety Translational Research Centre, Institute of Global Health Innovation, Department of Surgery & Cancer; London School of Hygiene and Tropical Medicine Faculty of Infectious and Tropical Diseases, Clinical Research Department<br>Neves, Ana Luisa; Imperial College London, Patient Safety Translational Research Centre, Institute of Global Health Innovation, Department of Surgery & Cancer; University of Porto, Center for Health Technology and Services Research / Department of Community Medicine, Health Information and Decision<br>Clarke, Jonathan; Imperial College London, Patient Safety Translational Research Centre, Institute of Global Health Innovation, Department of Surgery & Cancer; Imperial College London, Centre for Mathematics of Precision Healthcare<br>Ashrafian, Hutan; Imperial College London, Patient Safety Translational Research Centre, Institute of Global Health Innovation, Department of Surgery & Cancer<br>Darzi, Ara; Imperial College London, Patient Safety Translational Research Centre, Institute of Global Health Innovation, Department of Surgery & Cancer
**Primary Subject Heading**:	Health informatics
Secondary Subject Heading:	Health policy, Public health, Health services research
Keywords:	Health informatics < BIOTECHNOLOGY & BIOINFORMATICS, Telemedicine < BIOTECHNOLOGY & BIOINFORMATICS, Information technology < BIOTECHNOLOGY & BIOINFORMATICS, Information management < BIOTECHNOLOGY & BIOINFORMATICS, HEALTH SERVICES ADMINISTRATION & MANAGEMENT, Health policy < HEALTH SERVICES ADMINISTRATION & MANAGEMENT

SCHOLARONE™
Manuscripts

I, the Submitting Author has the right to grant and does grant on behalf of all authors of the Work (as defined in the below author licence), an exclusive licence and/or a non-exclusive licence for contributions from authors who are: i) UK Crown employees; ii) where BMJ has agreed a CC-BY licence shall apply, and/or iii) in accordance with the terms applicable for US Federal Government officers or employees acting as part of their official duties; on a worldwide, perpetual, irrevocable, royalty-free basis to BMJ Publishing Group Ltd ("BMJ") its licensees and where the relevant Journal is co-owned by BMJ to the co-owners of the Journal, to publish the Work in this journal and any other BMJ products and to exploit all rights, as set out in our licence.

The Submitting Author accepts and understands that any supply made under these terms is made by BMJ to the Submitting Author unless you are acting as an employee on behalf of your employer or a postgraduate student of an affiliated institution which is paying any applicable article publishing charge ("APC") for Open Access articles. Where the Submitting Author wishes to make the Work available on an Open Access basis (and intends to pay the relevant APC), the terms of reuse of such Open Access shall be governed by a Creative Commons licence – details of these licences and which Creative Commons licence will apply to this Work are set out in our licence referred to above.

Other than as permitted in any relevant BMJ Author's Self Archiving Policies, I confirm this Work has not been accepted for publication elsewhere, is not being considered for publication elsewhere and does not duplicate material already published. I confirm all authors consent to publication of this Work and authorise the granting of this licence.

For peer review only

Electronic Health Records, Interoperability, and Patient Safety in Health Systems of High-Income Countries: A Systematic Review Protocol

Edmond Li[1], Ana Luisa Neves[1,2], Jonathan Clarke[1,3], Hutan Ashrafian[1], Ara Darzi[1]
[1] Imperial NIHR Patient Safety Translational Research Centre (PSTRC), Institute of Global Health Innovation (IGHI), Imperial College London, London, United Kingdom
[2] Department of Community Medicine, Information and Health Decision Sciences (MEDCIDS) / Center for Health Technology and Services Research (CINTESIS), University of Porto, Porto, Portugal.
[3] Centre for Mathematics of Precision Healthcare, Imperial College London, London, United Kingdom

For peer review only

Corresponding author
Name: Edmond Li
Email: edmond.li19@imperial.ac.uk
Address: Institute of Global Health Innovation, Room 1035/7, QEQM Wing, St Mary's Campus, London W2 1NY
Keywords [MeSH terms]: electronic health records, electronic medical records, computerised patient records, health information technology, health information exchanges, interoperability, patient safety, patient harm, adverse events, health outcomes
Word count: 2723

Abstract

Introduction

The availability and routine use of electronic health records (EHRs) have become commonplace in healthcare systems of many high-income countries. While there is an ever-growing body of literature pertaining to EHR use, evidence surrounding the importance of EHR interoperability and its impact on patient safety remains less clear. There is therefore a need and opportunity to evaluate the evidence available regarding this relationship so as to better inform health informatics development and policies in the years to come.

Objective

This systematic review aims to evaluate the impact of EHR interoperability on patient safety in health systems of high-income countries.

Methods and analysis

A systematic literature review will be conducted via a computerised search through four databases: PubMed, Embase, HMIC, and PsycInfo for relevant articles published between 2010 and 2020. Outcomes of interest will include: impact on patient safety, and the broader effects on health systems. Quality of the randomised quantitative studies will be assessed using Cochrane Risk of Bias Tool. Non-randomised papers will be evaluated with the Risk of Bias In Non-Randomised Studies - of Interventions (ROBINS-I) tool. Drummond's Checklist will be utilised for publications pertaining to economic evaluation. The National Institute for Health and Care Excellence (NICE) quality appraisal checklist will be used to assess qualitative studies. A narrative synthesis will be conducted for included studies, and the body of evidence will be summarised in a summary of findings table.

Ethics and dissemination

This review will summarise published studies with non-identifiable data and therefore does not require ethical approval. This protocol complies with the Preferred Reporting Items for Systematic Review and Meta-Analyses Protocols guidelines. Findings will be disseminated through preprints, open access peer-reviewed publication, and conference presentations. Patients or members of the public were not involved in the design of this study.

Article Summary

Strengths and limitations of this study

Strengths

- Inclusion of quantitative, qualitative, and mixed-methods studies can provide a comprehensive overview of the multitude of ways in which interoperable EHRs may affect patient safety and health systems.
- The proposed review attempts to answer a pragmatic question which is integral to influencing future health informatics development and policies.

Limitations

- The heterogeneity of methods and outcomes assessed may obscure the true effect interoperable EHRs have had on patient safety in the clinical setting.

For peer review only - http://bmjopen.bmj.com/site/about/guidelines.xhtml

- Potential small sample size in subgroup analyses, may negatively impact the statistical power in quantitative data synthesis.
- Limiting the search strategy to English-only publications may not capture studies exploring the EHR interoperability experiences of health systems in non-English speaking countries.

PROSPERO registration number [CRD42020209285]

This is an open access article distributed in accordance with the Creative Commons Attribution 4.0 Unported (CC BY 4.0) license, which permits others to copy, redistribute, remix, transform and build upon this work for any purpose, provided the original work is properly cited, a link to the licence is given, and indication of whether changes were made. See: https://creativecommons.org/licenses/by/4.0/.

For peer review only

Introduction

Electronic health records (EHRs) have become an integral part of modern healthcare since their initial mainstream implementation in the mid-late 2000s through the passing of the Health Information Technology for Economic and Clinical Health Act (HITECH) in the United States and the NHS National Programme for IT initiative (NPfIT) in England (1–4). From the documentation & retrieval of patient records and the prescription of medications, to coordinating complex care plans between different healthcare providers and electronic billing, EHRs fulfil a multitude of roles for both clinicians and patients alike (5–9).

In order to achieve EHR's full potential, it is critical to improve interoperability - i.e., "*the ability of health information systems to work together within and across organisation boundaries in order to advance effective delivery of healthcare for individuals and communities*" (10). The lack of universal interoperability is often cited as one of the many significant shortcomings of EHRs currently in use, resulting in duplication in healthcare costs, increased clinician workload fatigue, and poses a potential risk to patient safety (2). This is especially problematic for patient populations with chronic conditions, polypharmacy, and multiple comorbidities who are reliant upon effective patient information sharing via EHRs to facilitate their care (11).

Poor EHR interoperability is detrimental to patient safety and costly for health systems. Its consequences range from increased risks of medication errors, fragmentation of patient data, to iatrogenic harm resulting from redundant testing, and additional healthcare expenditure (12–17). In the fragmented EHR landscape of the United Kingdom, measuring the effect poor EHR interoperability has in the National Health Service (NHS), remain challenging (18). Although there is a growing body of literature investigating areas such as the facilitators and barriers to EHR greater adoption, technical capabilities, and usability (19,20), no systematic review has been conducted exploring specifically the problem of interoperability amongst the assortment of EHRs in use, how it affects patient safety, and ultimately the financial cost savings lost to health systems.

In a recent systematic review by Dobrow *et al*. assessing the effects of EHR and HIT interoperability on health systems, 130 publications were included, with the majority being studies conducted in the United States, utilised quantitative methods, and focussed primarily in acute healthcare settings. The authors noted the use of interoperable EHRs had a positive impact on outcomes measures such as quality of care and productivity (19). However, in domains such as stakeholder engagement, performance & reliability, security & privacy, information quality, and ease of use, the benefits of interoperable EHRs was less clear (19). Amongst the 130 publications, 17 were reviews with the majority directed at exploring facilitators & barriers to EHR implementation, and the general benefits and impact of EHR use. While this review did focus on studies pertaining to the topic of interoperable EHRs, this was done so only from a broad perspective and included studies exploring a wide range of outcomes related to the effects of EHR on healthcare rather than specifically on their implications to patient safety.

In another review by Hersh *et al.*, the authors explored how health information exchange (HIE) affected health systems on a variety of domains, including costs, healthcare utilisation, health

For peer review only - http://bmjopen.bmj.com/site/about/guidelines.xhtml

outcomes, healthcare worker attitudes, and sustainability. Despite the widespread routinely use of HIE, the authors described a general lack of robust evidence on the quality, costs, efficiency, usage, and sustainability (21). However, there was some evidence demonstrating HIEs being associated with reduced utilisation and costs in emergency care settings despite methodological issues being present in many of the included publications (21). Although this review was ambitious in the wide scope of interest regarding the effects of HIE use, patient safety was not a primary topic of focus. Another limitation of this study was that it only contained US-based publications, and thus findings lack generalisability internationally to other health systems in high-income countries (HIC) which are both organised, financed differently.

Research aim

The overall aim of this literature review is to explore how EHR interoperability impacts patient safety, in the context of health systems in HICs. The results generated will aim to inform healthcare policymakers and help shape more effective EHR system implementation and modernisation efforts in the coming years.

Methods and analysis

Search strategy

A computerised search of the literature published in the last 10 years (2010 to 2020) search will be performed on PubMed/Medline, Embase, Cumulative Index to Nursing and Allied Health Literature (CINAHL), Health Management Information Consortium (HMIC), and PsycInfo. This publication timeframe was chosen as it coincides with the mainstream implementation of EHRs in several HIC healthcare systems such as Kaiser Permanente in the US, and thus would select for the most up to date, relevant evidence concerning EHR interoperability and patient safety challenges faced by healthcare systems today to be included (22,23). The list of search strings used will include both free text and controlled terms, whenever supported (Table 1) and will be iteratively refined in consultation with the Imperial College St. Mary's campus medical librarian.

Grey literature sources will also be searched, including registrations in the International Prospective Register of Systematic Reviews, reports of relevant stakeholder organisations (NHS England, American Medical Informatics Association (AMIA), eHealth at WHO, and conference proceedings (last 5 years) of several related conferences (AMIA, MedInfo, Medicine 2.0, Medicine X)), in order to identify possible additional studies that meet the inclusion criteria.

The search has also been restricted to HIC and articles published in English only.

Table 1: Concepts and database search terms

**Electronic health records**		**Interoperability**		**Patient safety**
• Electronic health records<br>• Electronic medical records<br>• Computerised medical records systems	**AND**	• Interoperability<br>• Health information interoperability<br>• Systems integration	**AND**	• Patient safety<br>• Patient adj1 incident*<br>• Adverse adj1 event*<br>• Patient adj1 outcome*

For peer review only

For peer review only - http://bmjopen.bmj.com/site/about/guidelines.xhtml

• Health information exchange / HIE<br>• Health information technology / HIT<br>• Hospital information systems<br>• Medical informatics<br>• Medical records linkage				• Patient adj1 harm<br>• Risk management

Study selection criteria

A summary of the population, intervention, comparison, outcomes, and type of studies being considered is provided in **Table 2**. This systematic review will focus on studies performed in high-income countries and published in English only. Studies assessing the impact of EHR interoperability will be included. Interventions will include EHR systems interoperable with other health information technology systems both within and across healthcare facilities, as well as those used in tertiary and community settings. The primary outcomes to be considered in this review will be safety outcomes, including adverse events/incidents, safety-related patient experiences, and health outcomes. In addition, secondary outcomes would include studies exploring the broader impact of interoperable EHRs on health systems such as cost effectiveness and clinical culture amongst healthcare providers on the topic Quantitative, qualitative, and mixed methods studies will be included. Reference lists of the selected articles will also be screened for papers which may have been missed by the initial database search but still meet the eligibility criteria.

Table 2: PICO inclusion criteria

Population	High-income countries utilising electronic health records
Intervention	EHRs with interoperability
Comparison	Usual care (i.e., existing baseline of interoperability)
Outcome	Impact on patient safety and quality of care

Screening

Articles to be included will be screened by two independent reviewers, following the process described in the Preferred Reporting Items for Systematic Reviews and Meta-Analyses (PRISMA) flow diagram (24). The initial screening will be done by the first reviewer based on the publication titles, followed by a second screening based on the abstracts. Included abstracts will then be fully reviewed by two independent researchers to produce a unified selection of articles to be included in this review. Cohen's kappa will be calculated to ensure inter-rater agreement and consistency in the selection of studies to be included (25,26). Any disagreements will be resolved by consensus; if a Cohen's kappa value of less than 0.6 is reported, the discrepancies will be addressed through discussions with a more experienced third investigator.

For peer review only - http://bmjopen.bmj.com/site/about/guidelines.xhtml

For peer review only

Data extraction

Data extraction will be performed using a standardised extraction table for each of the two investigators to summarise the characteristics and findings of each included study, including name of the first author, year of publication, study design, number of participants, retention rates, setting characteristics, outcome measures, and main results. The content of the two summary tables will then be aggregated and reviewed once more by both investigators, with any disagreements being solved by the third senior investigator.

Quality assessment

The quality of randomised controlled trials and cluster randomised trials will be assessed using the Cochrane Risk of Bias Tool (27), and the quality of non-randomised intervention studies (i.e., case control, cohort, quasi-experimental) will be appraised using the 'Risk of Bias In Non-Randomised Studies - of Interventions' (ROBINS-I) tool (28). For cost-effectiveness studies, the Drummond's checklist for assessing economic evaluations will be used (29). The National Institute for Health and Care Excellence (NICE) quality appraisal checklist will be utilised to assess the selected qualitative studies (30). Two independent reviewers will score the selected studies and any disagreements will be resolved by a third person. A risk of bias table along with an overall, collective bias narrative will be produced to summarise the biases of outcomes observed amongst the evaluated studies.

Narrative synthesis, subgroup analysis, and meta-analysis

A narrative synthesis will be performed for all studies included in this systematic review to summarise any salient findings observed (31).

In quantitative studies with homogenous or comparable outcome measures, whenever possible, continuous and dichotomous outcomes will be pooled together in a meta-analysis. If possible, effect sizes will be transformed in a common metric (Hedges' g – the bias-corrected standardised difference in means) and classified as positive when in favour of the intervention. Heterogeneity will be assessed using I^2 and the presence of publication bias will be evaluated using a funnel plot and the Duval and Tweedie's trim and fill method (32).

For both qualitative and quantitative studies that report comparable outcomes, a subgroup analysis based on clinical settings (e.g., primary vs. secondary healthcare settings) will be conducted to explore any patterns or relationships ascertained from the data. Through a standardised spreadsheet shared amongst the reviewers, the body of evidence will be organised in two separate Summary of Findings tables (for both qualitative and quantitative studies) in accordance to the 'Grading of Recommendations Assessment, Development and Evaluation' (GRADE) criteria (33).

Patient and public involvement

This systematic literature review saw no direct participation by patients or the public during the design of this study. However, this study was designed following a series of structured interviews with patients regarding their experience of attending multiple institutions for hospital care (34). As this literature review will be used to form the basis for subsequent studies exploring the topic including ones involving patients, findings from this review will be shared with patient

For peer review only - http://bmjopen.bmj.com/site/about/guidelines.xhtml

research groups to gain feedback and encourage further discourse surrounding the topic of EHR interoperability and patient safety.

Amendments

Any amendments to this protocol will be documented with reference to saved searches and analysis methods, which will be recorded in bibliographic databases, Mendeley and Excel templates for data collection and synthesis.

Discussion

One of the primary strengths stemming from the almost exploratory nature of this systematic review is the ability to generate a succinct, comprehensive appraisal of the best evidence currently available regarding how EHR interoperability impacts patient care and safety. By publishing this review protocol beforehand, we demonstrate a clear, robust, and transparent approach to aggregating the anticipated assortment of literature on the subject in question.

There are also some limitations to be acknowledged. By restricting the inclusion criteria to publications made English only, this could potentially exclude relevant papers pertaining to interoperable EHR systems in non-English healthcare settings. However, this is expected to be minimal as the majority of the papers concerning this topic published from the United States and European countries and are primarily done so in English journals. It must also be noted that both the heterogeneity of measures and outcomes evaluated, as well as the potentially reduced number of studies in subgroup analyses, may negatively influence the statistical power in data synthesis, and may preclude pooling of data as a meta-analysis. With such diverse means of measuring and assessing the effects of EHR interoperability, this will likely make comparisons between studies difficult and may obscure the true measure of effect EHR interoperability has had in the clinical setting. To mitigate this risk, outcomes will be grouped whenever possible, and summarised as a narrative synthesis. However, this can also represent a strength, as it will provide a comprehensive overview on the subject, capitalising on various research methodologies and providing novel insights on the impact of interoperable EHR systems on patient safety.

Acknowledgments

We would like to thank Michael Gainsford (Library Manager and Liaison Librarian at Imperial College London) for his support and guidance provided to improve the composition of the search terms and procedural aspects of the overall search strategy.

Authors' contributions

Conception and design of the work: ECL, ALN, and JC wrote the manuscript. HA, and AD provided critical revision of drafts for important intellectual content. All authors provided input into drafts of the manuscript and agree on the contents of the final version.

Funding statement

This research was supported through the Imperial College National Institute for Health Research (NIHR) Patient Safety Translational Research Centre (PSTRC) and the Imperial College Biomedical Research Centre (BRC). JC acknowledges support from the Wellcome Trust

For peer review only - http://bmjopen.bmj.com/site/about/guidelines.xhtml

[215938/Z/19/Z]. However, the funder/sponsor has had no role in development and drafting of this protocol.

Competing Interests

The authors declare that there are no competing interests.

For peer review only

For peer review only - http://bmjopen.bmj.com/site/about/guidelines.xhtml

References

1. Colicchio TK, Cimino JJ, Del Fiol G. Unintended consequences of nationwide electronic health record adoption: Challenges and opportunities in the post-meaningful use era. *Journal of Medical Internet Research*. [Online] 2019;21(6): 1–9. Available from: doi:10.2196/13313
2. Roman LC, Ancker JS, Johnson SB, Senathirajah Y. Navigation in the electronic health record: A review of the safety and usability literature. *Journal of Biomedical Informatics*. [Online] Elsevier Inc.; 2017;67: 69–79. Available from: doi:10.1016/j.jbi.2017.01.005
3. Justinia T. The UK's National Programme for IT: Why was it dismantled? *Health Services Management Research*. [Online] 2017;30(1): 2–9. Available from: doi:10.1177/0951484816662492
4. Wachter RM. Making IT work : harnessing the power of health IT to improve care in England. *National Advisory Group on Health Information Technology in England*. [Online] 2016; 71. Available from: https://www.gov.uk/government/uploads/system/uploads/attachment_data/file/550866/Wachter_Review_Accessible.pdf%0Ahttps://assets.publishing.service.gov.uk/government/uploads/system/uploads/attachment_data/file/550866/Wachter_Review_Accessible.pdf
5. Kruse CS, Kristof C, Jones B, Mitchell E, Martinez A. Barriers to Electronic Health Record Adoption: a Systematic Literature Review. *Journal of Medical Systems*. [Online] Journal of Medical Systems; 2016;40(12). Available from: doi:10.1007/s10916-016-0628-9
6. Ayaad O, Alloubani A, ALhajaa EA, Farhan M, Abuseif S, Al Hroub A, et al. The role of electronic medical records in improving the quality of health care services: Comparative study. *International Journal of Medical Informatics*. [Online] Elsevier; 2019;127(April): 63–67. Available from: doi:10.1016/j.ijmedinf.2019.04.014
7. Evans RS. Electronic Health Records: Then, Now, and in the Future. *Yearbook of Medical Informatics*. [Online] 2016;25(S 01): S48–S61. Available from: doi:10.15265/IYS-2016-s006
8. Gagnon MP, Payne-Gagnon J, Breton E, Fortin JP, Khoury L, Dolovich L, et al. Adoption of electronic personal health records in Canada: Perceptions of stakeholders. *International Journal of Health Policy and Management*. [Online] 2016;5(7): 425–433. Available from: doi:10.15171/ijhpm.2016.36
9. Campanella P, Lovato E, Marone C, Fallacara L, Mancuso A, Ricciardi W, et al. The impact of electronic health records on healthcare quality: a systematic review and meta-analysis. *The European Journal of Public Health*. [Online] 2016;26(1): 60–64. Available from: doi:10.1093/eurpub/ckv122
10. Reis ZSN, Maia TA, Marcolino MS, Becerra-Posada F, Novillo-Ortiz D, Ribeiro ALP. Is There Evidence of Cost Benefits of Electronic Medical Records, Standards, or Interoperability in Hospital Information Systems? Overview of Systematic Reviews. *JMIR Medical Informatics*. [Online] 2017;5(3): e26. Available from: doi:10.2196/medinform.7400
11. World Health Organization (WHO). *eHealth in the Region of the Americas: breaking down the barriers to implementation*. [Online] 2016. Available from: http://iris.paho.org/xmlui/bitstream/handle/123456789/31286/9789275119259-eng.pdf?sequence=6&isAllowed=y
12. Zaheer S, Pimentel SD, Simmons KD, Kuo LE, Datta J, Williams N, et al. Comparing

For peer review only

International and United States Undergraduate Medical Education and Surgical Outcomes Using a Refined Balance Matching Methodology. *Annals of Surgery*. [Online] 2017; Available from: doi:10.1097/SLA.0000000000001878
13. Everson J, Adler-Milstein J. Gaps in health information exchange between hospitals that treat many shared patients. *Journal of the American Medical Informatics Association*. [Online] 2018;25(9): 1114–1121. Available from: doi:10.1093/jamia/ocy089
14. Thompson MP, Graetz I. Hospital adoption of interoperability functions. *Healthcare*. [Online] Elsevier Inc.; 2019;7(3): 100347. Available from: doi:10.1016/j.hjdsi.2018.12.001
15. Tapuria A, Kalra D, Kobayashi S. Contribution of clinical archetypes, and the challenges, towards achieving semantic interoperability for EHRs. *Healthcare Informatics Research*. [Online] 2013;19(4): 286–292. Available from: doi:10.4258/hir.2013.19.4.286
16. Warren LR, Clarke JM, Arora S, Darzi AW. Improving data sharing between acute hospitals in England: an overview of health record system distribution and retrospective observational analysis of inter-hospital transitions of care. *BMJ Open*. [Online] 2019;9(12): e031637. Available from: doi:10.1136/bmjopen-2019-031637
17. Clarke JM, Warren LR, Arora S, Barahona M, Darzi AW. Guiding interoperable electronic health records through patient-sharing networks. *npj Digital Medicine*. [Online] Springer US; 2018;1(1): 1–6. Available from: doi:10.1038/s41746-018-0072-y
18. McAlearney AS, Sieck C, Hefner J, Robbins J, Huerta TR. Facilitating Ambulatory Electronic Health Record System Implementation: Evidence from a Qualitative Study. *BioMed Research International*. [Online] 2013;2013: 1–9. Available from: doi:10.1155/2013/629574
19. Dobrow MJ, Bytautas JP, Tharmalingam S, Hagens S. Interoperable Electronic Health Records and Health Information Exchanges: Systematic Review. *JMIR Medical Informatics*. [Online] 2019;7(2): e12607. Available from: doi:10.2196/12607
20. Zahabi M, Kaber DB, Swangnetr M. Usability and Safety in Electronic Medical Records Interface Design: A Review of Recent Literature and Guideline Formulation. *Human Factors*. [Online] 2015;57(5): 805–834. Available from: doi:10.1177/0018720815576827
21. Hersh WR, Totten AM, Eden KB, Devine B, Gorman P, Kassakian SZ, et al. Outcomes From Health Information Exchange: Systematic Review and Future Research Needs. *JMIR Medical Informatics*. [Online] 2015;3(4): e39. Available from: doi:10.2196/medinform.5215
22. Wheatley B. Transforming care delivery through health information technology. *The Permanente journal*. [Online] Winter; 2013;17(1): 81–86. Available from: doi:10.7812/TPP/12-030 [Accessed: 20th January 2021]
23. Chen C, Permanente K. The Kaiser Permanente Electronic Health Record: Transforming And Streamlining Modalities Of Care. 2009; Available from: doi:10.1377/hlthaff.28.2.323 [Accessed: 22nd January 2021]
24. Moher D, Liberati A, Tetzlaff J, Altman DG, Altman D, Antes G, et al. Preferred reporting items for systematic reviews and meta-analyses: The PRISMA statement. *PLoS Medicine*. [Online] 2009;6(7). Available from: doi:10.1371/journal.pmed.1000097
25. Landis JR, Koch GG. The Measurement of Observer Agreement for Categorical Data. *Biometrics*. [Online] 1977;33(1): 159. Available from: doi:10.2307/2529310
26. McHugh ML. Interrater reliability: the kappa statistic. *Biochemia medica*. 2012;22(3): 276–282.

For peer review only - http://bmjopen.bmj.com/site/about/guidelines.xhtml

27. Sterne JAC, Savović J, Page MJ, Elbers RG, Blencowe NS, Boutron I, et al. RoB 2: A revised tool for assessing risk of bias in randomised trials. *The BMJ*. [Online] 2019;366(August). Available from: doi:10.1136/bmj.l4898

28. Sterne JA, Hernán MA, Reeves BC, Savović J, Berkman ND, Viswanathan M, et al. ROBINS-I: A tool for assessing risk of bias in non-randomised studies of interventions. *BMJ (Online)*. [Online] 2016;355: 4–10. Available from: doi:10.1136/bmj.i4919

29. Drummond MF, Brien BJ, Torrance GW, Stoddart GL. *Methods for the Economic Evaluation of Health Care Programmes*.. 2nd ed. Oxford University Press; 1997.

30. *Methods for the development of NICE public health guidance*. [Online]. 3rd n. National Institute for Clinical Excellence (NICE); 2018. Available from: https://www.nice.org.uk/process/pmg4/resources/methods-for-the-development-of-nice-public-health-guidance-third-edition-pdf-2007967445701

31. Popay, J., Roberts, H., Sowden, A., Petticrew, M., Arai, L., Rodgers, M., ... & Duffy S. Guidance on the conduct of narrative synthesis in systematic reviews. A product from the ESRC methods programme. 2006;(April 2006): 1–92.

32. Duval S, Tweedie R. Trim and fill: A simple funnel-plot-based method of testing and adjusting for publication bias in meta-analysis. *Biometrics*. [Online] Biometric Society; 2000;56(2): 455–463. Available from: doi:10.1111/j.0006-341X.2000.00455.x [Accessed: 20th January 2021]

33. Guyatt GH, Oxman AD, Vist GE, Kunz R, Falck-Ytter Y, Alonso-Coello P, et al. GRADE: an emerging consensus on rating quality of evidence and strength of recommendations. *BMJ*. [Online] 2008;336(7650): 924–926. Available from: doi:10.1136/bmj.39489.470347.AD

34. Warren LR, Harrison M, Arora S, Darzi A. Working with patients and the public to design an electronic health record interface: A qualitative mixed-methods study. *BMC Medical Informatics and Decision Making*. [Online] BMC Medical Informatics and Decision Making; 2019;19(1): 1–8. Available from: doi:10.1186/s12911-019-0993-7

For peer review only

PRISMA-P (Preferred Reporting Items for Systematic review and Meta-Analysis Protocols) 2015 checklist: recommended items to address in a systematic review protocol*

Section and topic	Item No.	Checklist item	Reported on Page No.
**ADMINISTRATIVE INFORMATION**			
Title:			
Identification	1a	Identify the report as a protocol of a systematic review	1
Update	1b	If the protocol is for an update of a previous systematic review, identify as such	N/A
Registration	2	If registered, provide the name of the registry (such as PROSPERO) and registration number	3
Authors:			
Contact	3a	Provide name, institutional affiliation, e-mail address of all protocol authors; provide physical mailing address of corresponding author	1
Contributions	3b	Describe contributions of protocol authors and identify the guarantor of the review	1
Amendments	4	If the protocol represents an amendment of a previously completed or published protocol, identify as such and list changes; otherwise, state plan for documenting important protocol amendments	N/A
Support:			
Sources	5a	Indicate sources of financial or other support for the review	8
Sponsor	5b	Provide name for the review funder and/or sponsor	8
Role of sponsor or funder	5c	Describe roles of funder(s), sponsor(s), and/or institution(s), if any, in developing the protocol	8
**INTRODUCTION**			
Rationale	6	Describe the rationale for the review in the context of what is already known	4-5
Objectives	7	Provide an explicit statement of the question(s) the review will address with reference to participants, interventions, comparators, and outcomes (PICO)	5
**METHODS**			
Eligibility criteria	8	Specify the study characteristics (such as PICO, study design, setting, time frame) and report characteristics (such as years considered, language, publication status) to be used as criteria for eligibility for the review	5-6
Information sources	9	Describe all intended information sources (such as electronic databases, contact with study authors, trial registers or other grey literature sources) with planned dates of coverage	5
Search strategy	10	Present draft of search strategy to be used for at least one electronic database, including planned limits, such that it could be repeated	5

For peer review only

Study records:			
Data management	11a	Describe the mechanism(s) that will be used to manage records and data throughout the review	6-7
Selection process	11b	State the process that will be used for selecting studies (such as two independent reviewers) through each phase of the review (that is, screening, eligibility and inclusion in meta-analysis)	6
Data collection process	11c	Describe planned method of extracting data from reports (such as piloting forms, done independently, in duplicate), any processes for obtaining and confirming data from investigators	6-7
Data items	12	List and define all variables for which data will be sought (such as PICO items, funding sources), any pre-planned data assumptions and simplifications	6
Outcomes and prioritization	13	List and define all outcomes for which data will be sought, including prioritization of main and additional outcomes, with rationale	6
Risk of bias in individual studies	14	Describe anticipated methods for assessing risk of bias of individual studies, including whether this will be done at the outcome or study level, or both; state how this information will be used in data synthesis	6-7
Data synthesis	15a	Describe criteria under which study data will be quantitatively synthesised	6-7
	15b	If data are appropriate for quantitative synthesis, describe planned summary measures, methods of handling data and methods of combining data from studies, including any planned exploration of consistency (such as I^2, Kendall's τ)	7
	15c	Describe any proposed additional analyses (such as sensitivity or subgroup analyses, meta-regression)	-
	15d	If quantitative synthesis is not appropriate, describe the type of summary planned	7
Meta-bias(es)	16	Specify any planned assessment of meta-bias(es) (such as publication bias across studies, selective reporting within studies)	6-7
Confidence in cumulative evidence	17	Describe how the strength of the body of evidence will be assessed (such as GRADE)	7

*** It is strongly recommended that this checklist be read in conjunction with the PRISMA-P Explanation and Elaboration (cite when available) for important clarification on the items. Amendments to a review protocol should be tracked and dated.** **The copyright for PRISMA-P (including checklist) is held by the PRISMA-P Group and is distributed under a Creative Commons Attribution Licence 4.0.**

From: Shamseer L, Moher D, Clarke M, Ghersi D, Liberati A, Petticrew M, Shekelle P, Stewart L, PRISMA-P Group. Preferred reporting items for systematic review and meta-analysis protocols (PRISMA-P) 2015: elaboration and explanation. BMJ. 2015 Jan 2;349(jan02 1):g7647.

For peer review only

BMJ Open

Electronic Health Records, Interoperability, and Patient Safety in Health Systems of High-Income Countries: A Systematic Review Protocol

Journal:	*BMJ Open*
Manuscript ID	bmjopen-2020-044941.R2
Article Type:	Protocol
Date Submitted by the Author:	27-May-2021
Complete List of Authors:	Li, Edmond; Imperial College London, Patient Safety Translational Research Centre, Institute of Global Health Innovation, Department of Surgery & Cancer; London School of Hygiene and Tropical Medicine Faculty of Infectious and Tropical Diseases, Clinical Research Department<br>Clarke, Jonathan; Imperial College London, Patient Safety Translational Research Centre, Institute of Global Health Innovation, Department of Surgery & Cancer; Imperial College London, Centre for Mathematics of Precision Healthcare<br>Neves, Ana Luisa; Imperial College London, Patient Safety Translational Research Centre, Institute of Global Health Innovation, Department of Surgery & Cancer; University of Porto, Center for Health Technology and Services Research / Department of Community Medicine, Health Information and Decision<br>Ashrafian, Hutan; Imperial College London, Patient Safety Translational Research Centre, Institute of Global Health Innovation, Department of Surgery & Cancer<br>Darzi, Ara; Imperial College London, Patient Safety Translational Research Centre, Institute of Global Health Innovation, Department of Surgery & Cancer
**Primary Subject Heading**:	Health informatics
Secondary Subject Heading:	Health policy, Public health, Health services research
Keywords:	Health informatics < BIOTECHNOLOGY & BIOINFORMATICS, Telemedicine < BIOTECHNOLOGY & BIOINFORMATICS, Information technology < BIOTECHNOLOGY & BIOINFORMATICS, Information management < BIOTECHNOLOGY & BIOINFORMATICS, HEALTH SERVICES ADMINISTRATION & MANAGEMENT, Health policy < HEALTH SERVICES ADMINISTRATION & MANAGEMENT

SCHOLARONE™
Manuscripts

I, the Submitting Author has the right to grant and does grant on behalf of all authors of the Work (as defined in the below author licence), an exclusive licence and/or a non-exclusive licence for contributions from authors who are: i) UK Crown employees; ii) where BMJ has agreed a CC-BY licence shall apply, and/or iii) in accordance with the terms applicable for US Federal Government officers or employees acting as part of their official duties; on a worldwide, perpetual, irrevocable, royalty-free basis to BMJ Publishing Group Ltd ("BMJ") its licensees and where the relevant Journal is co-owned by BMJ to the co-owners of the Journal, to publish the Work in this journal and any other BMJ products and to exploit all rights, as set out in our licence.

The Submitting Author accepts and understands that any supply made under these terms is made by BMJ to the Submitting Author unless you are acting as an employee on behalf of your employer or a postgraduate student of an affiliated institution which is paying any applicable article publishing charge ("APC") for Open Access articles. Where the Submitting Author wishes to make the Work available on an Open Access basis (and intends to pay the relevant APC), the terms of reuse of such Open Access shall be governed by a Creative Commons licence – details of these licences and which Creative Commons licence will apply to this Work are set out in our licence referred to above.

Other than as permitted in any relevant BMJ Author's Self Archiving Policies, I confirm this Work has not been accepted for publication elsewhere, is not being considered for publication elsewhere and does not duplicate material already published. I confirm all authors consent to publication of this Work and authorise the granting of this licence.

For peer review only

Electronic Health Records, Interoperability, and Patient Safety in Health Systems of High-Income Countries: A Systematic Review Protocol

Edmond Li[1], Jonathan Clarke[1,3], Ana Luisa Neves[1,2], Hutan Ashrafian[1], Ara Darzi[1]
[1] Imperial NIHR Patient Safety Translational Research Centre (PSTRC), Institute of Global Health Innovation (IGHI), Imperial College London, London, United Kingdom
[2] Department of Community Medicine, Information and Health Decision Sciences (MEDCIDS) / Center for Health Technology and Services Research (CINTESIS), University of Porto, Porto, Portugal.
[3] Centre for Mathematics of Precision Healthcare, Imperial College London, London, United Kingdom

For peer review only

Corresponding author
Name: Edmond Li
Email: edmond.li19@imperial.ac.uk
Address: Institute of Global Health Innovation, Room 1035/7, QEQM Wing, St Mary's Campus, London W2 1NY
Keywords [MeSH terms]: electronic health records, electronic medical records, computerised patient records, health information technology, health information exchanges, interoperability, patient safety, patient harm, adverse events, health outcomes
Word count: 2752

For peer review only - http://bmjopen.bmj.com/site/about/guidelines.xhtml

Abstract

Introduction

The availability and routine use of electronic health records (EHRs) have become commonplace in healthcare systems of many high-income countries. While there is an ever-growing body of literature pertaining to EHR use, evidence surrounding the importance of EHR interoperability and its impact on patient safety remains less clear. There is therefore a need and opportunity to evaluate the evidence available regarding this relationship so as to better inform health informatics development and policies in the years to come.

Objective

This systematic review aims to evaluate the impact of EHR interoperability on patient safety in health systems of high-income countries.

Methods and analysis

A systematic literature review will be conducted via a computerised search through four databases: PubMed, Embase, HMIC, and PsycInfo for relevant articles published between 2010 and 2020. Outcomes of interest will include: impact on patient safety, and the broader effects on health systems. Quality of the randomised quantitative studies will be assessed using Cochrane Risk of Bias Tool. Non-randomised papers will be evaluated with the Risk of Bias In Non-Randomised Studies - of Interventions (ROBINS-I) tool. Drummond's Checklist will be utilised for publications pertaining to economic evaluation. The National Institute for Health and Care Excellence (NICE) quality appraisal checklist will be used to assess qualitative studies. A narrative synthesis will be conducted for included studies, and the body of evidence will be summarised in a summary of findings table.

Ethics and dissemination

This review will summarise published studies with non-identifiable data and therefore does not require ethical approval. This protocol complies with the Preferred Reporting Items for Systematic Review and Meta-Analyses Protocols guidelines. Findings will be disseminated through preprints, open access peer-reviewed publication, and conference presentations. Patients or members of the public were not involved in the design of this study.

For peer review only

Article Summary

Strengths and limitations of this study

Strengths

- Inclusion of quantitative, qualitative, and mixed-methods studies can provide a comprehensive overview of the multitude of ways in which interoperable EHRs may affect patient safety and health systems.
- The proposed review attempts to answer a pragmatic question which is integral to influencing future health informatics development and policies.

Limitations

- The heterogeneity of methods and outcomes assessed may obscure the true effect interoperable EHRs have had on patient safety in the clinical setting.

For peer review only - http://bmjopen.bmj.com/site/about/guidelines.xhtml

- Potential small sample size in subgroup analyses, may negatively impact the statistical power in quantitative data synthesis.
- Limiting the search strategy to English-only publications may not capture studies exploring the EHR interoperability experiences of health systems in non-English speaking countries.

PROSPERO registration number [CRD42020209285]

This is an open access article distributed in accordance with the Creative Commons Attribution 4.0 Unported (CC BY 4.0) license, which permits others to copy, redistribute, remix, transform and build upon this work for any purpose, provided the original work is properly cited, a link to the licence is given, and indication of whether changes were made. See: https://creativecommons.org/licenses/by/4.0/.

For peer review only

For peer review only - http://bmjopen.bmj.com/site/about/guidelines.xhtml

Introduction

Electronic health records (EHRs) have become an integral part of modern healthcare since their initial mainstream implementation in the mid-late 2000s through the passing of the Health Information Technology for Economic and Clinical Health Act (HITECH) in the United States and the NHS National Programme for IT initiative (NPfIT) in England (1–4). From the documentation & retrieval of patient records and the prescription of medications, to coordinating complex care plans between different healthcare providers and electronic billing, EHRs fulfil a multitude of roles for both clinicians and patients alike (5–9).

In order to achieve EHR's full potential, it is critical to improve interoperability - i.e., "*the ability of health information systems to work together within and across organisation boundaries in order to advance effective delivery of healthcare for individuals and communities*" (10). The lack of universal interoperability is often cited as one of the many significant shortcomings of EHRs currently in use, resulting in duplication in healthcare costs, increased clinician workload fatigue, and poses a potential risk to patient safety (2). This is especially problematic for patient populations with chronic conditions, polypharmacy, and multiple comorbidities who are reliant upon effective patient information sharing via EHRs to facilitate their care (11).

Poor EHR interoperability is detrimental to patient safety and costly for health systems. Its consequences range from increased risks of medication errors, fragmentation of patient data, to iatrogenic harm resulting from redundant testing, and additional healthcare expenditure (12–17). In the fragmented EHR landscape of the United Kingdom, measuring the effect poor EHR interoperability has in the National Health Service (NHS), remain challenging (18). Although there is a growing body of literature investigating areas such as the facilitators and barriers to EHR greater adoption, technical capabilities, and usability (19,20), no systematic review has been conducted exploring specifically the problem of interoperability amongst the assortment of EHRs in use, how it affects patient safety, and ultimately the financial cost savings lost to health systems.

In a recent systematic review by Dobrow *et al*. assessing the effects of EHR and HIT interoperability on health systems, 130 publications were included, with the majority being studies conducted in the United States, utilised quantitative methods, and focussed primarily on acute healthcare settings. The authors noted the use of interoperable EHRs had a positive impact on outcomes measures such as quality of care and productivity (19). However, in domains such as stakeholder engagement, performance & reliability, security & privacy, information quality, and ease of use, the benefits of interoperable EHRs was less clear (19). Amongst the 130 publications, 17 were reviews with the majority directed at exploring facilitators & barriers to EHR implementation, and the general benefits and impact of EHR use. While this review did focus on studies pertaining to the topic of interoperable EHRs, this was done so only from a broad perspective and included studies exploring a wide range of outcomes related to the effects of EHR on healthcare rather than specifically on their implications to patient safety.

In another review by Hersh *et al.*, the authors explored how health information exchange (HIE) affected health systems on a variety of domains, including costs, healthcare utilisation, health

For peer review only - http://bmjopen.bmj.com/site/about/guidelines.xhtml

outcomes, healthcare worker attitudes, and sustainability. Despite the widespread routinely use of HIE, the authors described a general lack of robust evidence on the quality, costs, efficiency, usage, and sustainability (21). However, there was some evidence demonstrating HIEs being associated with reduced utilisation and costs in emergency care settings despite methodological issues being present in many of the included publications (21). Although this review was ambitious in the wide scope of interest regarding the effects of HIE use, patient safety was not a primary topic of focus. Another limitation of this study was that it only contained US-based publications, and thus findings lack generalisability internationally to other health systems in high-income countries (HIC) which are both organised, financed differently.

Research aim

The overall aim of this literature review is to explore how EHR interoperability impacts patient safety, in the context of health systems in HICs. The results generated will aim to inform healthcare policymakers and help shape more effective EHR system implementation and modernisation efforts in the coming years.

Methods and analysis

Search strategy

A computerised search of the literature published in the last 10 years (2010 to 2020) search will be performed on PubMed/Medline, Embase, Cumulative Index to Nursing and Allied Health Literature (CINAHL), Health Management Information Consortium (HMIC), and PsycInfo. This publication timeframe was chosen as it coincides with the mainstream implementation of EHRs in several HIC healthcare systems such as Kaiser Permanente in the US, and thus would select for the most up to date, relevant evidence concerning EHR interoperability and patient safety challenges faced by healthcare systems today to be included (22,23). The list of search strings used will include both free text and controlled terms, whenever supported (Table 1) and will be iteratively refined in consultation with the Imperial College St. Mary's campus medical librarian.

Grey literature sources will also be searched, including registrations in the International Prospective Register of Systematic Reviews, reports of relevant stakeholder organisations (NHS England, American Medical Informatics Association (AMIA), eHealth at WHO, and conference proceedings (last 5 years) of several related conferences (AMIA, MedInfo, Medicine 2.0, Medicine X)), in order to identify possible additional studies that meet the inclusion criteria.

The search has also been restricted to HIC and articles published in English only.

Table 1: Concepts and database search terms

**Electronic health records**		**Interoperability**		**Patient safety**
• Electronic health records<br>• Electronic medical records<br>• Computerised medical records systems	**AND**	• Interoperability<br>• Health information interoperability<br>• Systems integration	**AND**	• Patient safety<br>• Patient adj1 incident*<br>• Adverse adj1 event*<br>• Patient adj1 outcome*

For peer review only - http://bmjopen.bmj.com/site/about/guidelines.xhtml

• Health information exchange / HIE<br>• Health information technology / HIT<br>• Hospital information systems<br>• Medical informatics<br>• Medical records linkage				• Patient adj1 harm<br>• Risk management

Study selection criteria

A summary of the population, intervention, comparison, outcomes, and type of studies being considered is provided in **Table 2**. This systematic review will focus on studies performed in high-income countries and published in English only. High-income countries will be defined in accordance with the World Bank's definition of "*countries where the gross national income (GNI) per capita is higher than $12,536 USD*" (24). Studies assessing the impact of EHR interoperability will be included. Interventions will include EHR systems interoperable with other health information technology systems both within and across healthcare facilities, as well as those used in tertiary and community settings. The primary outcomes to be considered in this review will be safety outcomes, including adverse events/incidents, safety-related patient experiences, and health outcomes. In addition, secondary outcomes would include studies exploring the broader impact of interoperable EHRs on health systems such as cost effectiveness and clinical culture amongst healthcare providers on the topic Quantitative, qualitative, and mixed methods studies will be included. Reference lists of the selected articles will also be screened for papers which may have been missed by the initial database search but still meet the eligibility criteria.

Table 2: PICO inclusion criteria

Population	High-income countries utilising electronic health records
Intervention	EHRs with interoperability
Comparison	Usual care (i.e., existing baseline of interoperability)
Outcome	Impact on patient safety and quality of care

Screening

Articles to be included will be screened by two independent reviewers, following the process described in the Preferred Reporting Items for Systematic Reviews and Meta-Analyses (PRISMA) flow diagram (25). The initial screening will be done by the first reviewer based on the publication titles, followed by a second screening based on the abstracts. Included abstracts will then be fully reviewed by two independent researchers to produce a unified selection of articles to be included in this review. Cohen's kappa will be calculated to ensure inter-rater agreement and consistency in the selection of studies to be included (26,27). Any disagreements

For peer review only

For peer review only - http://bmjopen.bmj.com/site/about/guidelines.xhtml

will be resolved by consensus; if a Cohen's kappa value of less than 0.6 is reported, the discrepancies will be addressed through discussions with a more experienced third investigator.

Data extraction

Data extraction will be performed using a standardised extraction table for each of the two investigators to summarise the characteristics and findings of each included study, including name of the first author, year of publication, study design, number of participants, retention rates, setting characteristics, outcome measures, and main results. The content of the two summary tables will then be aggregated and reviewed once more by both investigators, with any disagreements being solved by the third senior investigator.

Quality assessment

The quality of randomised controlled trials and cluster randomised trials will be assessed using the Cochrane Risk of Bias Tool (28), and the quality of non-randomised intervention studies (i.e., case control, cohort, quasi-experimental) will be appraised using the 'Risk of Bias In Non-Randomised Studies - of Interventions' (ROBINS-I) tool (29). For cost-effectiveness studies, the Drummond's checklist for assessing economic evaluations will be used (30). The National Institute for Health and Care Excellence (NICE) quality appraisal checklist will be utilised to assess the selected qualitative studies (31). Two independent reviewers will score the selected studies and any disagreements will be resolved by a third person. A risk of bias table along with an overall, collective bias narrative will be produced to summarise the biases of outcomes observed amongst the evaluated studies.

Narrative synthesis, subgroup analysis, and meta-analysis

A narrative synthesis will be performed for all studies included in this systematic review to summarise any salient findings observed (32).

In quantitative studies with homogenous or comparable outcome measures, whenever possible, continuous and dichotomous outcomes will be pooled together in a meta-analysis. If possible, effect sizes will be transformed in a common metric (Hedges' g – the bias-corrected standardised difference in means) and classified as positive when in favour of the intervention. Heterogeneity will be assessed using I^2 and the presence of publication bias will be evaluated using a funnel plot and the Duval and Tweedie's trim and fill method (33).

For both qualitative and quantitative studies that report comparable outcomes, a subgroup analysis based on clinical settings (e.g., primary vs. secondary healthcare settings) will be conducted to explore any patterns or relationships ascertained from the data. Through a standardised spreadsheet shared amongst the reviewers, the body of evidence will be organised in two separate Summary of Findings tables (for both qualitative and quantitative studies) in accordance to the 'Grading of Recommendations Assessment, Development and Evaluation' (GRADE) criteria (34).

Patient and public involvement

This systematic literature review saw no direct participation by patients or the public during the design of this study. However, this study was designed following a series of structured interviews with patients regarding their experience of attending multiple institutions for hospital

For peer review only - http://bmjopen.bmj.com/site/about/guidelines.xhtml

care (35). As this literature review will be used to form the basis for subsequent studies exploring the topic including ones involving patients, findings from this review will be shared with patient research groups to gain feedback and encourage further discourse surrounding the topic of EHR interoperability and patient safety.

Amendments

Any amendments to this protocol will be documented with reference to saved searches and analysis methods, which will be recorded in bibliographic databases, Mendeley and Excel templates for data collection and synthesis.

Discussion

One of the primary strengths stemming from the almost exploratory nature of this systematic review is the ability to generate a succinct, comprehensive appraisal of the best evidence currently available regarding how EHR interoperability impacts patient care and safety. By publishing this review protocol beforehand, we demonstrate a clear, robust, and transparent approach to aggregating the anticipated assortment of literature on the subject in question.

There are also some limitations to be acknowledged. By restricting the inclusion criteria to publications made in English only, this could potentially exclude relevant papers pertaining to interoperable EHR systems in non-English healthcare settings. However, this is expected to be minimal as the majority of the papers concerning this topic published from the United States and European countries and are primarily done so in English journals. It must also be noted that both the heterogeneity of measures and outcomes evaluated, as well as the potentially reduced number of studies in subgroup analyses, may negatively influence the statistical power in data synthesis, and may preclude pooling of data as a meta-analysis. With such diverse means of measuring and assessing the effects of EHR interoperability, this will likely make comparisons between studies difficult and may obscure the true measure of effect EHR interoperability has had in the clinical setting. To mitigate this risk, outcomes will be grouped whenever possible, and summarised as a narrative synthesis. However, this can also represent a strength, as it will provide a comprehensive overview on the subject, capitalising on various research methodologies and providing novel insights on the impact of interoperable EHR systems on patient safety.

Acknowledgments

We would like to thank Michael Gainsford (Library Manager and Liaison Librarian at Imperial College London) for his support and guidance provided to improve the composition of the search terms and procedural aspects of the overall search strategy.

Authors' contributions

Conception and design of the work: EL, ALN, and JC wrote the manuscript. HA, and AD provided critical revision of drafts for important intellectual content. All authors provided input into drafts of the manuscript and agree on the contents of the final version.

Funding statement

This research was supported through the Imperial College National Institute for Health Research (NIHR) Patient Safety Translational Research Centre (PSTRC) and the Imperial College

For peer review only - http://bmjopen.bmj.com/site/about/guidelines.xhtml

Biomedical Research Centre (BRC). JC acknowledges support from the Wellcome Trust [215938/Z/19/Z]. However, the funder/sponsor has had no role in development and drafting of this protocol.

Competing Interests

The authors declare that there are no competing interests.

For peer review only

For peer review only - http://bmjopen.bmj.com/site/about/guidelines.xhtml

References

1. Colicchio TK, Cimino JJ, Del Fiol G. Unintended consequences of nationwide electronic health record adoption: Challenges and opportunities in the post-meaningful use era. *Journal of Medical Internet Research*. [Online] 2019;21(6): 1–9. Available from: doi:10.2196/13313
2. Roman LC, Ancker JS, Johnson SB, Senathirajah Y. Navigation in the electronic health record: A review of the safety and usability literature. *Journal of Biomedical Informatics*. [Online] Elsevier Inc.; 2017;67: 69–79. Available from: doi:10.1016/j.jbi.2017.01.005
3. Justinia T. The UK's National Programme for IT: Why was it dismantled? *Health Services Management Research*. [Online] 2017;30(1): 2–9. Available from: doi:10.1177/0951484816662492
4. Wachter RM. Making IT work : harnessing the power of health IT to improve care in England. *National Advisory Group on Health Information Technology in England*. [Online] 2016; 71. Available from: https://www.gov.uk/government/uploads/system/uploads/attachment_data/file/550866/Wachter_Review_Accessible.pdf%0Ahttps://assets.publishing.service.gov.uk/government/uploads/system/uploads/attachment_data/file/550866/Wachter_Review_Accessible.pdf
5. Kruse CS, Kristof C, Jones B, Mitchell E, Martinez A. Barriers to Electronic Health Record Adoption: a Systematic Literature Review. *Journal of Medical Systems*. [Online] Journal of Medical Systems; 2016;40(12). Available from: doi:10.1007/s10916-016-0628-9
6. Ayaad O, Alloubani A, ALhajaa EA, Farhan M, Abuseif S, Al Hroub A, et al. The role of electronic medical records in improving the quality of health care services: Comparative study. *International Journal of Medical Informatics*. [Online] Elsevier; 2019;127(April): 63–67. Available from: doi:10.1016/j.ijmedinf.2019.04.014
7. Evans RS. Electronic Health Records: Then, Now, and in the Future. *Yearbook of Medical Informatics*. [Online] 2016;25(S 01): S48–S61. Available from: doi:10.15265/IYS-2016-s006
8. Gagnon MP, Payne-Gagnon J, Breton E, Fortin JP, Khoury L, Dolovich L, et al. Adoption of electronic personal health records in Canada: Perceptions of stakeholders. *International Journal of Health Policy and Management*. [Online] 2016;5(7): 425–433. Available from: doi:10.15171/ijhpm.2016.36
9. Campanella P, Lovato E, Marone C, Fallacara L, Mancuso A, Ricciardi W, et al. The impact of electronic health records on healthcare quality: a systematic review and meta-analysis. *The European Journal of Public Health*. [Online] 2016;26(1): 60–64. Available from: doi:10.1093/eurpub/ckv122
10. Reis ZSN, Maia TA, Marcolino MS, Becerra-Posada F, Novillo-Ortiz D, Ribeiro ALP. Is There Evidence of Cost Benefits of Electronic Medical Records, Standards, or Interoperability in Hospital Information Systems? Overview of Systematic Reviews. *JMIR Medical Informatics*. [Online] 2017;5(3): e26. Available from: doi:10.2196/medinform.7400
11. World Health Organization (WHO). *eHealth in the Region of the Americas: breaking down the barriers to implementation*. [Online] 2016. Available from: http://iris.paho.org/xmlui/bitstream/handle/123456789/31286/9789275119259-eng.pdf?sequence=6&isAllowed=y
12. Zaheer S, Pimentel SD, Simmons KD, Kuo LE, Datta J, Williams N, et al. Comparing

For peer review only - http://bmjopen.bmj.com/site/about/guidelines.xhtml

International and United States Undergraduate Medical Education and Surgical Outcomes Using a Refined Balance Matching Methodology. *Annals of Surgery*. [Online] 2017; Available from: doi:10.1097/SLA.0000000000001878
13. Everson J, Adler-Milstein J. Gaps in health information exchange between hospitals that treat many shared patients. *Journal of the American Medical Informatics Association*. [Online] 2018;25(9): 1114–1121. Available from: doi:10.1093/jamia/ocy089
14. Thompson MP, Graetz I. Hospital adoption of interoperability functions. *Healthcare*. [Online] Elsevier Inc.; 2019;7(3): 100347. Available from: doi:10.1016/j.hjdsi.2018.12.001
15. Tapuria A, Kalra D, Kobayashi S. Contribution of clinical archetypes, and the challenges, towards achieving semantic interoperability for EHRs. *Healthcare Informatics Research*. [Online] 2013;19(4): 286–292. Available from: doi:10.4258/hir.2013.19.4.286
16. Warren LR, Clarke JM, Arora S, Darzi AW. Improving data sharing between acute hospitals in England: an overview of health record system distribution and retrospective observational analysis of inter-hospital transitions of care. *BMJ Open*. [Online] 2019;9(12): e031637. Available from: doi:10.1136/bmjopen-2019-031637
17. Clarke JM, Warren LR, Arora S, Barahona M, Darzi AW. Guiding interoperable electronic health records through patient-sharing networks. *npj Digital Medicine*. [Online] Springer US; 2018;1(1): 1–6. Available from: doi:10.1038/s41746-018-0072-y
18. McAlearney AS, Sieck C, Hefner J, Robbins J, Huerta TR. Facilitating Ambulatory Electronic Health Record System Implementation: Evidence from a Qualitative Study. *BioMed Research International*. [Online] 2013;2013: 1–9. Available from: doi:10.1155/2013/629574
19. Dobrow MJ, Bytautas JP, Tharmalingam S, Hagens S. Interoperable Electronic Health Records and Health Information Exchanges: Systematic Review. *JMIR Medical Informatics*. [Online] 2019;7(2): e12607. Available from: doi:10.2196/12607
20. Zahabi M, Kaber DB, Swangnetr M. Usability and Safety in Electronic Medical Records Interface Design: A Review of Recent Literature and Guideline Formulation. *Human Factors*. [Online] 2015;57(5): 805–834. Available from: doi:10.1177/0018720815576827
21. Hersh WR, Totten AM, Eden KB, Devine B, Gorman P, Kassakian SZ, et al. Outcomes From Health Information Exchange: Systematic Review and Future Research Needs. *JMIR Medical Informatics*. [Online] 2015;3(4): e39. Available from: doi:10.2196/medinform.5215
22. Wheatley B. Transforming care delivery through health information technology. *The Permanente journal*. [Online] Winter; 2013;17(1): 81–86. Available from: doi:10.7812/TPP/12-030 [Accessed: 20th January 2021]
23. Chen C, Permanente K. The Kaiser Permanente Electronic Health Record: Transforming And Streamlining Modalities Of Care. 2009; Available from: doi:10.1377/hlthaff.28.2.323 [Accessed: 22nd January 2021]
24. World Bank. *World Bank Country and Lending Groups – World Bank Data Help Desk*. [Online] The World Bank. p. 1–8. Available from: https://datahelpdesk.worldbank.org/knowledgebase/articles/906519 [Accessed: 26th May 2021]
25. Moher D, Liberati A, Tetzlaff J, Altman DG, Altman D, Antes G, et al. Preferred reporting items for systematic reviews and meta-analyses: The PRISMA statement. *PLoS Medicine*. [Online] 2009;6(7). Available from: doi:10.1371/journal.pmed.1000097

For peer review only - http://bmjopen.bmj.com/site/about/guidelines.xhtml

26. Landis JR, Koch GG. The Measurement of Observer Agreement for Categorical Data. *Biometrics*. [Online] 1977;33(1): 159. Available from: doi:10.2307/2529310
27. McHugh ML. Interrater reliability: the kappa statistic. *Biochemia medica*. 2012;22(3): 276–282.
28. Sterne JAC, Savović J, Page MJ, Elbers RG, Blencowe NS, Boutron I, et al. RoB 2: A revised tool for assessing risk of bias in randomised trials. *The BMJ*. [Online] 2019;366(August). Available from: doi:10.1136/bmj.l4898
29. Sterne JA, Hernán MA, Reeves BC, Savović J, Berkman ND, Viswanathan M, et al. ROBINS-I: A tool for assessing risk of bias in non-randomised studies of interventions. *BMJ (Online)*. [Online] 2016;355: 4–10. Available from: doi:10.1136/bmj.i4919
30. Drummond MF, Brien BJ, Torrance GW, Stoddart GL. *Methods for the Economic Evaluation of Health Care Programmes*.. 2nd ed. Oxford University Press; 1997.
31. *Methods for the development of NICE public health guidance*. [Online]. 3rd n. National Institute for Clinical Excellence (NICE); 2018. Available from: https://www.nice.org.uk/process/pmg4/resources/methods-for-the-development-of-nice-public-health-guidance-third-edition-pdf-2007967445701
32. Popay, J., Roberts, H., Sowden, A., Petticrew, M., Arai, L., Rodgers, M., ... & Duffy S. Guidance on the conduct of narrative synthesis in systematic reviews. A product from the ESRC methods programme. 2006;(April 2006): 1–92.
33. Duval S, Tweedie R. Trim and fill: A simple funnel-plot-based method of testing and adjusting for publication bias in meta-analysis. *Biometrics*. [Online] Biometric Society; 2000;56(2): 455–463. Available from: doi:10.1111/j.0006-341X.2000.00455.x [Accessed: 20th January 2021]
34. Guyatt GH, Oxman AD, Vist GE, Kunz R, Falck-Ytter Y, Alonso-Coello P, et al. GRADE: an emerging consensus on rating quality of evidence and strength of recommendations. *BMJ*. [Online] 2008;336(7650): 924–926. Available from: doi:10.1136/bmj.39489.470347.AD
35. Warren LR, Harrison M, Arora S, Darzi A. Working with patients and the public to design an electronic health record interface: A qualitative mixed-methods study. *BMC Medical Informatics and Decision Making*. [Online] BMC Medical Informatics and Decision Making; 2019;19(1): 1–8. Available from: doi:10.1186/s12911-019-0993-7

For peer review only

For peer review only - http://bmjopen.bmj.com/site/about/guidelines.xhtml

PRISMA-P (Preferred Reporting Items for Systematic review and Meta-Analysis Protocols) 2015 checklist: recommended items to address in a systematic review protocol*

Section and topic	Item No.	Checklist item	Reported on Page No.
**ADMINISTRATIVE INFORMATION**			
Title:			
Identification	1a	Identify the report as a protocol of a systematic review	1
Update	1b	If the protocol is for an update of a previous systematic review, identify as such	N/A
Registration	2	If registered, provide the name of the registry (such as PROSPERO) and registration number	3
Authors:			
Contact	3a	Provide name, institutional affiliation, e-mail address of all protocol authors; provide physical mailing address of corresponding author	1
Contributions	3b	Describe contributions of protocol authors and identify the guarantor of the review	1
Amendments	4	If the protocol represents an amendment of a previously completed or published protocol, identify as such and list changes; otherwise, state plan for documenting important protocol amendments	N/A
Support:			
Sources	5a	Indicate sources of financial or other support for the review	8
Sponsor	5b	Provide name for the review funder and/or sponsor	8
Role of sponsor or funder	5c	Describe roles of funder(s), sponsor(s), and/or institution(s), if any, in developing the protocol	8
**INTRODUCTION**			
Rationale	6	Describe the rationale for the review in the context of what is already known	4-5
Objectives	7	Provide an explicit statement of the question(s) the review will address with reference to participants, interventions, comparators, and outcomes (PICO)	5
**METHODS**			
Eligibility criteria	8	Specify the study characteristics (such as PICO, study design, setting, time frame) and report characteristics (such as years considered, language, publication status) to be used as criteria for eligibility for the review	5-6
Information sources	9	Describe all intended information sources (such as electronic databases, contact with study authors, trial registers or other grey literature sources) with planned dates of coverage	5
Search strategy	10	Present draft of search strategy to be used for at least one electronic database, including planned limits, such that it could be repeated	5

For peer review only

Study records:			
Data management	11a	Describe the mechanism(s) that will be used to manage records and data throughout the review	6-7
Selection process	11b	State the process that will be used for selecting studies (such as two independent reviewers) through each phase of the review (that is, screening, eligibility and inclusion in meta-analysis)	6
Data collection process	11c	Describe planned method of extracting data from reports (such as piloting forms, done independently, in duplicate), any processes for obtaining and confirming data from investigators	6-7
Data items	12	List and define all variables for which data will be sought (such as PICO items, funding sources), any pre-planned data assumptions and simplifications	6
Outcomes and prioritization	13	List and define all outcomes for which data will be sought, including prioritization of main and additional outcomes, with rationale	6
Risk of bias in individual studies	14	Describe anticipated methods for assessing risk of bias of individual studies, including whether this will be done at the outcome or study level, or both; state how this information will be used in data synthesis	6-7
Data synthesis	15a	Describe criteria under which study data will be quantitatively synthesised	6-7
	15b	If data are appropriate for quantitative synthesis, describe planned summary measures, methods of handling data and methods of combining data from studies, including any planned exploration of consistency (such as I^2, Kendall's τ)	7
	15c	Describe any proposed additional analyses (such as sensitivity or subgroup analyses, meta-regression)	-
	15d	If quantitative synthesis is not appropriate, describe the type of summary planned	7
Meta-bias(es)	16	Specify any planned assessment of meta-bias(es) (such as publication bias across studies, selective reporting within studies)	6-7
Confidence in cumulative evidence	17	Describe how the strength of the body of evidence will be assessed (such as GRADE)	7

*** It is strongly recommended that this checklist be read in conjunction with the PRISMA-P Explanation and Elaboration (cite when available) for important clarification on the items. Amendments to a review protocol should be tracked and dated. The copyright for PRISMA-P (including checklist) is held by the PRISMA-P Group and is distributed under a Creative Commons Attribution Licence 4.0.**

From: Shamseer L, Moher D, Clarke M, Ghersi D, Liberati A, Petticrew M, Shekelle P, Stewart L, PRISMA-P Group. Preferred reporting items for systematic review and meta-analysis protocols (PRISMA-P) 2015: elaboration and explanation. BMJ. 2015 Jan 2;349(jan02 1):g7647.

For peer review only

BMJ Open

Electronic Health Records, Interoperability, and Patient Safety in Health Systems of High-Income Countries: A Systematic Review Protocol

Journal:	*BMJ Open*
Manuscript ID	bmjopen-2020-044941.R3
Article Type:	Protocol
Date Submitted by the Author:	05-Jun-2021
Complete List of Authors:	Li, Edmond; Imperial College London, Patient Safety Translational Research Centre, Institute of Global Health Innovation, Department of Surgery & Cancer; London School of Hygiene and Tropical Medicine Faculty of Infectious and Tropical Diseases, Clinical Research Department<br>Clarke, Jonathan; Imperial College London, Patient Safety Translational Research Centre, Institute of Global Health Innovation, Department of Surgery & Cancer; Imperial College London, Centre for Mathematics of Precision Healthcare<br>Neves, Ana Luisa; Imperial College London, Patient Safety Translational Research Centre, Institute of Global Health Innovation, Department of Surgery & Cancer; University of Porto, Center for Health Technology and Services Research / Department of Community Medicine, Health Information and Decision<br>Ashrafian, Hutan; Imperial College London, Patient Safety Translational Research Centre, Institute of Global Health Innovation, Department of Surgery & Cancer<br>Darzi, Ara; Imperial College London, Patient Safety Translational Research Centre, Institute of Global Health Innovation, Department of Surgery & Cancer
**Primary Subject Heading**:	Health informatics
Secondary Subject Heading:	Health policy, Public health, Health services research
Keywords:	Health informatics < BIOTECHNOLOGY & BIOINFORMATICS, Telemedicine < BIOTECHNOLOGY & BIOINFORMATICS, Information technology < BIOTECHNOLOGY & BIOINFORMATICS, Information management < BIOTECHNOLOGY & BIOINFORMATICS, HEALTH SERVICES ADMINISTRATION & MANAGEMENT, Health policy < HEALTH SERVICES ADMINISTRATION & MANAGEMENT

SCHOLARONE™
Manuscripts

I, the Submitting Author has the right to grant and does grant on behalf of all authors of the Work (as defined in the below author licence), an exclusive licence and/or a non-exclusive licence for contributions from authors who are: i) UK Crown employees; ii) where BMJ has agreed a CC-BY licence shall apply, and/or iii) in accordance with the terms applicable for US Federal Government officers or employees acting as part of their official duties; on a worldwide, perpetual, irrevocable, royalty-free basis to BMJ Publishing Group Ltd ("BMJ") its licensees and where the relevant Journal is co-owned by BMJ to the co-owners of the Journal, to publish the Work in this journal and any other BMJ products and to exploit all rights, as set out in our licence.

The Submitting Author accepts and understands that any supply made under these terms is made by BMJ to the Submitting Author unless you are acting as an employee on behalf of your employer or a postgraduate student of an affiliated institution which is paying any applicable article publishing charge ("APC") for Open Access articles. Where the Submitting Author wishes to make the Work available on an Open Access basis (and intends to pay the relevant APC), the terms of reuse of such Open Access shall be governed by a Creative Commons licence – details of these licences and which Creative Commons licence will apply to this Work are set out in our licence referred to above.

Other than as permitted in any relevant BMJ Author's Self Archiving Policies, I confirm this Work has not been accepted for publication elsewhere, is not being considered for publication elsewhere and does not duplicate material already published. I confirm all authors consent to publication of this Work and authorise the granting of this licence.

For peer review only

Electronic Health Records, Interoperability, and Patient Safety in Health Systems of High-Income Countries: A Systematic Review Protocol

Edmond Li[1], Jonathan Clarke[1,3], Ana Luisa Neves[1,2], Hutan Ashrafian[1], Ara Darzi[1]
[1] Imperial NIHR Patient Safety Translational Research Centre (PSTRC), Institute of Global Health Innovation (IGHI), Imperial College London, London, United Kingdom
[2] Department of Community Medicine, Information and Health Decision Sciences (MEDCIDS) / Center for Health Technology and Services Research (CINTESIS), University of Porto, Porto, Portugal.
[3] Centre for Mathematics of Precision Healthcare, Imperial College London, London, United Kingdom

For peer review only

Corresponding author
Name: Edmond Li
Email: edmond.li19@imperial.ac.uk
Address: Institute of Global Health Innovation, Room 1035/7, QEQM Wing, St Mary's Campus, London W2 1NY

Keywords [MeSH terms]: electronic health records, electronic medical records, computerised patient records, health information technology, health information exchanges, interoperability, patient safety, patient harm, adverse events, health outcomes

For peer review only - http://bmjopen.bmj.com/site/about/guidelines.xhtml

Abstract

Introduction

The availability and routine use of electronic health records (EHRs) have become commonplace in healthcare systems of many high-income countries. While there is an ever-growing body of literature pertaining to EHR use, evidence surrounding the importance of EHR interoperability and its impact on patient safety remains less clear. There is therefore a need and opportunity to evaluate the evidence available regarding this relationship so as to better inform health informatics development and policies in the years to come. This systematic review aims to evaluate the impact of EHR interoperability on patient safety in health systems of high-income countries.

Methods and analysis

A systematic literature review will be conducted via a computerised search through four databases: PubMed, Embase, HMIC, and PsycInfo for relevant articles published between 2010 and 2020. Outcomes of interest will include: impact on patient safety, and the broader effects on health systems. Quality of the randomised quantitative studies will be assessed using Cochrane Risk of Bias Tool. Non-randomised papers will be evaluated with the Risk of Bias In Non-Randomised Studies - of Interventions (ROBINS-I) tool. Drummond's Checklist will be utilised for publications pertaining to economic evaluation. The National Institute for Health and Care Excellence (NICE) quality appraisal checklist will be used to assess qualitative studies. A narrative synthesis will be conducted for included studies, and the body of evidence will be summarised in a summary of findings table.

Ethics and dissemination

This review will summarise published studies with non-identifiable data and thus does not require ethical approval. Findings will be disseminated through preprints, open access peer-reviewed publication, and conference presentations.

PROSPERO registration number [CRD42020209285]

This is an open access article distributed in accordance with the Creative Commons Attribution 4.0 Unported (CC BY 4.0) license, which permits others to copy, redistribute, remix, transform and build upon this work for any purpose, provided the original work is properly cited, a link to the licence is given, and indication of whether changes were made. See: https://creativecommons.org/licenses/by/4.0/.

Article Summary

Strengths and limitations of this study

Strengths

- Inclusion of quantitative, qualitative, and mixed-methods studies can provide a comprehensive overview of the multitude of ways in which interoperable EHRs may affect patient safety and health systems.
- Using robust methodology to examine the wealth of existing literature, the proposed systematic review attempts to answer a pragmatic question which is integral to future health informatics development and policies.

For peer review only

For peer review only - http://bmjopen.bmj.com/site/about/guidelines.xhtml

Limitations

- The heterogeneity of methods and outcomes assessed may obscure the true effect interoperable EHRs have had on patient safety.
- Potential small sample size in subgroup analyses, may negatively impact the statistical power in quantitative data synthesis.
- Limiting the search strategy to English-only publications may not capture studies exploring EHR experiences in non-English speaking countries.

For peer review only

For peer review only - http://bmjopen.bmj.com/site/about/guidelines.xhtml

Introduction

Electronic health records (EHRs) have become an integral part of modern healthcare since their initial mainstream implementation in the mid-late 2000s through the passing of the Health Information Technology for Economic and Clinical Health Act (HITECH) in the United States and the NHS National Programme for IT initiative (NPfIT) in England (1–4). From the documentation & retrieval of patient records and the prescription of medications, to coordinating complex care plans between different healthcare providers and electronic billing, EHRs fulfil a multitude of roles for both clinicians and patients alike (5–9).

In order to achieve EHR's full potential, it is critical to improve interoperability - i.e., "*the ability of health information systems to work together within and across organisation boundaries in order to advance effective delivery of healthcare for individuals and communities*" (10). The lack of universal interoperability is often cited as one of the many significant shortcomings of EHRs currently in use, resulting in duplication in healthcare costs, increased clinician workload fatigue, and poses a potential risk to patient safety (2). This is especially problematic for patient populations with chronic conditions, polypharmacy, and multiple comorbidities who are reliant upon effective patient information sharing via EHRs to facilitate their care (11).

Poor EHR interoperability is detrimental to patient safety and costly for health systems. Its consequences range from increased risks of medication errors, fragmentation of patient data, to iatrogenic harm resulting from redundant testing, and additional healthcare expenditure (12–17). In the fragmented EHR landscape of the United Kingdom, measuring the effect poor EHR interoperability has in the National Health Service (NHS), remain challenging (18). Although there is a growing body of literature investigating areas such as the facilitators and barriers to EHR greater adoption, technical capabilities, and usability (19,20), no systematic review has been conducted exploring specifically the problem of interoperability amongst the assortment of EHRs in use, how it affects patient safety, and ultimately the financial cost savings lost to health systems.

In a recent systematic review by Dobrow *et al*. assessing the effects of EHR and HIT interoperability on health systems, 130 publications were included, with the majority being studies conducted in the United States, utilised quantitative methods, and focussed primarily on acute healthcare settings. The authors noted the use of interoperable EHRs had a positive impact on outcomes measures such as quality of care and productivity (19). However, in domains such as stakeholder engagement, performance & reliability, security & privacy, information quality, and ease of use, the benefits of interoperable EHRs was less clear (19). Amongst the 130 publications, 17 were reviews with the majority directed at exploring facilitators & barriers to EHR implementation, and the general benefits and impact of EHR use. While this review did focus on studies pertaining to the topic of interoperable EHRs, this was done so only from a broad perspective and included studies exploring a wide range of outcomes related to the effects of EHR on healthcare rather than specifically on their implications to patient safety.

In another review by Hersh *et al*., the authors explored how health information exchange (HIE) affected health systems on a variety of domains, including costs, healthcare utilisation, health

For peer review only - http://bmjopen.bmj.com/site/about/guidelines.xhtml

outcomes, healthcare worker attitudes, and sustainability. Despite the widespread routinely use of HIE, the authors described a general lack of robust evidence on the quality, costs, efficiency, usage, and sustainability (21). However, there was some evidence demonstrating HIEs being associated with reduced utilisation and costs in emergency care settings despite methodological issues being present in many of the included publications (21). Although this review was ambitious in the wide scope of interest regarding the effects of HIE use, patient safety was not a primary topic of focus. Another limitation of this study was that it only contained US-based publications, and thus findings lack generalisability internationally to other health systems in high-income countries (HIC) which are both organised, financed differently.

Research aim

The overall aim of this literature review is to explore how EHR interoperability impacts patient safety, in the context of health systems in HICs. The results generated will aim to inform healthcare policymakers and help shape more effective EHR system implementation and modernisation efforts in the coming years.

Methods and analysis

Search strategy

A computerised search of the literature published in the last 10 years (2010 to 2020) search will be performed on PubMed/Medline, Embase, Cumulative Index to Nursing and Allied Health Literature (CINAHL), Health Management Information Consortium (HMIC), and PsycInfo. This publication timeframe was chosen as it coincides with the mainstream implementation of EHRs in several HIC healthcare systems such as Kaiser Permanente in the US, and thus would select for the most up to date, relevant evidence concerning EHR interoperability and patient safety challenges faced by healthcare systems today to be included (22,23). The list of search strings used will include both free text and controlled terms, whenever supported (Table 1) and will be iteratively refined in consultation with the Imperial College St. Mary's campus medical librarian. For a sample of the search strategy, please see Supplement 1.

Grey literature sources will also be searched, including registrations in the International Prospective Register of Systematic Reviews, reports of relevant stakeholder organisations (NHS England, American Medical Informatics Association (AMIA), eHealth at WHO, and conference proceedings (last 5 years) of several related conferences (AMIA, MedInfo, Medicine 2.0, Medicine X)), in order to identify possible additional studies that meet the inclusion criteria.

The search has also been restricted to HIC and articles published in English only.

Table 1: Concepts and database search terms

**Electronic health records**		**Interoperability**		**Patient safety**
• Electronic health records<br>• Electronic medical records	**AND**	• Interoperability<br>• Health information interoperability<br>• Systems integration	**AND**	• Patient safety<br>• Patient adj1 incident*<br>• Adverse adj1 event*

For peer review only - http://bmjopen.bmj.com/site/about/guidelines.xhtml

• Computerised medical records systems<br>• Health information exchange / HIE<br>• Health information technology / HIT<br>• Hospital information systems<br>• Medical informatics<br>• Medical records linkage				• Patient adj1 outcome*<br>• Patient adj1 harm<br>• Risk management

Study selection criteria

A summary of the population, intervention, comparison, outcomes, and type of studies being considered is provided in **Table 2**. This systematic review will focus on studies performed in high-income countries and published in English only. High-income countries will be defined in accordance with the World Bank's definition of "*countries where the gross national income (GNI) per capita is higher than $12,536 USD*" (24). Studies assessing the impact of EHR interoperability will be included. Interventions will include EHR systems interoperable with other health information technology systems both within and across healthcare facilities, as well as those used in tertiary and community settings. The primary outcomes to be considered in this review will be safety outcomes, including adverse events/incidents, safety-related patient experiences, and health outcomes. In addition, secondary outcomes would include studies exploring the broader impact of interoperable EHRs on health systems such as cost effectiveness and clinical culture amongst healthcare providers on the topic Quantitative, qualitative, and mixed methods studies will be included. Reference lists of the selected articles will also be screened for papers which may have been missed by the initial database search but still meet the eligibility criteria.

Table 2: PICO inclusion criteria

Population	High-income countries utilising electronic health records
Intervention	EHRs with interoperability
Comparison	Usual care (i.e., existing baseline of interoperability)
Outcome	Impact on patient safety and quality of care

Screening

Articles to be included will be screened by two independent reviewers, following the process described in the Preferred Reporting Items for Systematic Reviews and Meta-Analyses (PRISMA) flow diagram (25). The initial screening will be done by the first reviewer based on

For peer review only - http://bmjopen.bmj.com/site/about/guidelines.xhtml

For peer review only

the publication titles, followed by a second screening based on the abstracts. Included abstracts will then be fully reviewed by two independent researchers to produce a unified selection of articles to be included in this review. Cohen's kappa will be calculated to ensure inter-rater agreement and consistency in the selection of studies to be included (26,27). Any disagreements will be resolved by consensus; if a Cohen's kappa value of less than 0.6 is reported, the discrepancies will be addressed through discussions with a more experienced third investigator.

Data extraction

Data extraction will be performed using a standardised extraction table for each of the two investigators to summarise the characteristics and findings of each included study, including name of the first author, year of publication, study design, number of participants, retention rates, setting characteristics, outcome measures, and main results. The content of the two summary tables will then be aggregated and reviewed once more by both investigators, with any disagreements being solved by the third senior investigator.

Quality assessment

The quality of randomised controlled trials and cluster randomised trials will be assessed using the Cochrane Risk of Bias Tool (28), and the quality of non-randomised intervention studies (i.e., case control, cohort, quasi-experimental) will be appraised using the 'Risk of Bias In Non-Randomised Studies - of Interventions' (ROBINS-I) tool (29). For cost-effectiveness studies, the Drummond's checklist for assessing economic evaluations will be used (30). The National Institute for Health and Care Excellence (NICE) quality appraisal checklist will be utilised to assess the selected qualitative studies (31). Two independent reviewers will score the selected studies and any disagreements will be resolved by a third person. A risk of bias table along with an overall, collective bias narrative will be produced to summarise the biases of outcomes observed amongst the evaluated studies.

Narrative synthesis, subgroup analysis, and meta-analysis

A narrative synthesis will be performed for all studies included in this systematic review to summarise any salient findings observed (32).

In quantitative studies with homogenous or comparable outcome measures, whenever possible, continuous and dichotomous outcomes will be pooled together in a meta-analysis. If possible, effect sizes will be transformed in a common metric (Hedges' g – the bias-corrected standardised difference in means) and classified as positive when in favour of the intervention. Heterogeneity will be assessed using I^2 and the presence of publication bias will be evaluated using a funnel plot and the Duval and Tweedie's trim and fill method (33).

For both qualitative and quantitative studies that report comparable outcomes, a subgroup analysis based on clinical settings (e.g., primary vs. secondary healthcare settings) will be conducted to explore any patterns or relationships ascertained from the data. Through a standardised spreadsheet shared amongst the reviewers, the body of evidence will be organised in two separate Summary of Findings tables (for both qualitative and quantitative studies) in accordance to the 'Grading of Recommendations Assessment, Development and Evaluation' (GRADE) criteria (34).

For peer review only - http://bmjopen.bmj.com/site/about/guidelines.xhtml

Patient and public involvement

This systematic literature review saw no direct participation by patients or the public during the design of this study. However, this study was designed following a series of structured interviews with patients regarding their experience of attending multiple institutions for hospital care (35). As this literature review will be used to form the basis for subsequent studies exploring the topic including ones involving patients, findings from this review will be shared with patient research groups to gain feedback and encourage further discourse surrounding the topic of EHR interoperability and patient safety.

Amendments

Any amendments to this protocol will be documented with reference to saved searches and analysis methods, which will be recorded in bibliographic databases, Mendeley and Excel templates for data collection and synthesis.

Discussion

One of the primary strengths stemming from the almost exploratory nature of this systematic review is the ability to generate a succinct, comprehensive appraisal of the best evidence currently available regarding how EHR interoperability impacts patient care and safety. By publishing this review protocol beforehand, we demonstrate a clear, robust, and transparent approach to aggregating the anticipated assortment of literature on the subject in question.

There are also some limitations to be acknowledged. By restricting the inclusion criteria to publications made in English only, this could potentially exclude relevant papers pertaining to interoperable EHR systems in non-English healthcare settings. However, this is expected to be minimal as the majority of the papers concerning this topic published from the United States and European countries and are primarily done so in English journals. It must also be noted that both the heterogeneity of measures and outcomes evaluated, as well as the potentially reduced number of studies in subgroup analyses, may negatively influence the statistical power in data synthesis, and may preclude pooling of data as a meta-analysis. With such diverse means of measuring and assessing the effects of EHR interoperability, this will likely make comparisons between studies difficult and may obscure the true measure of effect EHR interoperability has had in the clinical setting. To mitigate this risk, outcomes will be grouped whenever possible, and summarised as a narrative synthesis. However, this can also represent a strength, as it will provide a comprehensive overview on the subject, capitalising on various research methodologies and providing novel insights on the impact of interoperable EHR systems on patient safety.

Ethics and Dissemination

This review will summarise published studies with non-identifiable data and therefore does not require ethical approval. This protocol complies with the Preferred Reporting Items for Systematic Review and Meta-Analyses Protocols guidelines. Findings will be disseminated through preprints, open access peer-reviewed publication, and conference presentations.

For peer review only - http://bmjopen.bmj.com/site/about/guidelines.xhtml

Acknowledgments

We would like to thank Michael Gainsford (Library Manager and Liaison Librarian at Imperial College London) for his support and guidance provided to improve the composition of the search terms and procedural aspects of the overall search strategy.

For peer review only

For peer review only - http://bmjopen.bmj.com/site/about/guidelines.xhtml

References

1. Colicchio TK, Cimino JJ, Del Fiol G. Unintended consequences of nationwide electronic health record adoption: Challenges and opportunities in the post-meaningful use era. *Journal of Medical Internet Research*. [Online] 2019;21(6): 1–9. Available from: doi:10.2196/13313
2. Roman LC, Ancker JS, Johnson SB, Senathirajah Y. Navigation in the electronic health record: A review of the safety and usability literature. *Journal of Biomedical Informatics*. [Online] Elsevier Inc.; 2017;67: 69–79. Available from: doi:10.1016/j.jbi.2017.01.005
3. Justinia T. The UK's National Programme for IT: Why was it dismantled? *Health Services Management Research*. [Online] 2017;30(1): 2–9. Available from: doi:10.1177/0951484816662492
4. Wachter RM. Making IT work : harnessing the power of health IT to improve care in England. *National Advisory Group on Health Information Technology in England*. [Online] 2016; 71. Available from: https://www.gov.uk/government/uploads/system/uploads/attachment_data/file/550866/Wachter_Review_Accessible.pdf%0Ahttps://assets.publishing.service.gov.uk/government/uploads/system/uploads/attachment_data/file/550866/Wachter_Review_Accessible.pdf
5. Kruse CS, Kristof C, Jones B, Mitchell E, Martinez A. Barriers to Electronic Health Record Adoption: a Systematic Literature Review. *Journal of Medical Systems*. [Online] Journal of Medical Systems; 2016;40(12). Available from: doi:10.1007/s10916-016-0628-9
6. Ayaad O, Alloubani A, ALhajaa EA, Farhan M, Abuseif S, Al Hroub A, et al. The role of electronic medical records in improving the quality of health care services: Comparative study. *International Journal of Medical Informatics*. [Online] Elsevier; 2019;127(April): 63–67. Available from: doi:10.1016/j.ijmedinf.2019.04.014
7. Evans RS. Electronic Health Records: Then, Now, and in the Future. *Yearbook of Medical Informatics*. [Online] 2016;25(S 01): S48–S61. Available from: doi:10.15265/IYS-2016-s006
8. Gagnon MP, Payne-Gagnon J, Breton E, Fortin JP, Khoury L, Dolovich L, et al. Adoption of electronic personal health records in Canada: Perceptions of stakeholders. *International Journal of Health Policy and Management*. [Online] 2016;5(7): 425–433. Available from: doi:10.15171/ijhpm.2016.36
9. Campanella P, Lovato E, Marone C, Fallacara L, Mancuso A, Ricciardi W, et al. The impact of electronic health records on healthcare quality: a systematic review and meta-analysis. *The European Journal of Public Health*. [Online] 2016;26(1): 60–64. Available from: doi:10.1093/eurpub/ckv122
10. Reis ZSN, Maia TA, Marcolino MS, Becerra-Posada F, Novillo-Ortiz D, Ribeiro ALP. Is There Evidence of Cost Benefits of Electronic Medical Records, Standards, or Interoperability in Hospital Information Systems? Overview of Systematic Reviews. *JMIR Medical Informatics*. [Online] 2017;5(3): e26. Available from: doi:10.2196/medinform.7400
11. World Health Organization (WHO). *eHealth in the Region of the Americas: breaking down the barriers to implementation*. [Online] 2016. Available from: http://iris.paho.org/xmlui/bitstream/handle/123456789/31286/9789275119259-eng.pdf?sequence=6&isAllowed=y
12. Zaheer S, Pimentel SD, Simmons KD, Kuo LE, Datta J, Williams N, et al. Comparing

For peer review only - http://bmjopen.bmj.com/site/about/guidelines.xhtml

International and United States Undergraduate Medical Education and Surgical Outcomes Using a Refined Balance Matching Methodology. *Annals of Surgery*. [Online] 2017; Available from: doi:10.1097/SLA.0000000000001878
13. Everson J, Adler-Milstein J. Gaps in health information exchange between hospitals that treat many shared patients. *Journal of the American Medical Informatics Association*. [Online] 2018;25(9): 1114–1121. Available from: doi:10.1093/jamia/ocy089
14. Thompson MP, Graetz I. Hospital adoption of interoperability functions. *Healthcare*. [Online] Elsevier Inc.; 2019;7(3): 100347. Available from: doi:10.1016/j.hjdsi.2018.12.001
15. Tapuria A, Kalra D, Kobayashi S. Contribution of clinical archetypes, and the challenges, towards achieving semantic interoperability for EHRs. *Healthcare Informatics Research*. [Online] 2013;19(4): 286–292. Available from: doi:10.4258/hir.2013.19.4.286
16. Warren LR, Clarke JM, Arora S, Darzi AW. Improving data sharing between acute hospitals in England: an overview of health record system distribution and retrospective observational analysis of inter-hospital transitions of care. *BMJ Open*. [Online] 2019;9(12): e031637. Available from: doi:10.1136/bmjopen-2019-031637
17. Clarke JM, Warren LR, Arora S, Barahona M, Darzi AW. Guiding interoperable electronic health records through patient-sharing networks. *npj Digital Medicine*. [Online] Springer US; 2018;1(1): 1–6. Available from: doi:10.1038/s41746-018-0072-y
18. McAlearney AS, Sieck C, Hefner J, Robbins J, Huerta TR. Facilitating Ambulatory Electronic Health Record System Implementation: Evidence from a Qualitative Study. *BioMed Research International*. [Online] 2013;2013: 1–9. Available from: doi:10.1155/2013/629574
19. Dobrow MJ, Bytautas JP, Tharmalingam S, Hagens S. Interoperable Electronic Health Records and Health Information Exchanges: Systematic Review. *JMIR Medical Informatics*. [Online] 2019;7(2): e12607. Available from: doi:10.2196/12607
20. Zahabi M, Kaber DB, Swangnetr M. Usability and Safety in Electronic Medical Records Interface Design: A Review of Recent Literature and Guideline Formulation. *Human Factors*. [Online] 2015;57(5): 805–834. Available from: doi:10.1177/0018720815576827
21. Hersh WR, Totten AM, Eden KB, Devine B, Gorman P, Kassakian SZ, et al. Outcomes From Health Information Exchange: Systematic Review and Future Research Needs. *JMIR Medical Informatics*. [Online] 2015;3(4): e39. Available from: doi:10.2196/medinform.5215
22. Wheatley B. Transforming care delivery through health information technology. *The Permanente journal*. [Online] Winter; 2013;17(1): 81–86. Available from: doi:10.7812/TPP/12-030 [Accessed: 20th January 2021]
23. Chen C, Permanente K. The Kaiser Permanente Electronic Health Record: Transforming And Streamlining Modalities Of Care. 2009; Available from: doi:10.1377/hlthaff.28.2.323 [Accessed: 22nd January 2021]
24. World Bank. *World Bank Country and Lending Groups – World Bank Data Help Desk*. [Online] The World Bank. p. 1–8. Available from: https://datahelpdesk.worldbank.org/knowledgebase/articles/906519 [Accessed: 26th May 2021]
25. Moher D, Liberati A, Tetzlaff J, Altman DG, Altman D, Antes G, et al. Preferred reporting items for systematic reviews and meta-analyses: The PRISMA statement. *PLoS Medicine*. [Online] 2009;6(7). Available from: doi:10.1371/journal.pmed.1000097

For peer review only - http://bmjopen.bmj.com/site/about/guidelines.xhtml

26. Landis JR, Koch GG. The Measurement of Observer Agreement for Categorical Data. *Biometrics*. [Online] 1977;33(1): 159. Available from: doi:10.2307/2529310

27. McHugh ML. Interrater reliability: the kappa statistic. *Biochemia medica*. 2012;22(3): 276–282.

28. Sterne JAC, Savović J, Page MJ, Elbers RG, Blencowe NS, Boutron I, et al. RoB 2: A revised tool for assessing risk of bias in randomised trials. *The BMJ*. [Online] 2019;366(August). Available from: doi:10.1136/bmj.l4898

29. Sterne JA, Hernán MA, Reeves BC, Savović J, Berkman ND, Viswanathan M, et al. ROBINS-I: A tool for assessing risk of bias in non-randomised studies of interventions. *BMJ (Online)*. [Online] 2016;355: 4–10. Available from: doi:10.1136/bmj.i4919

30. Drummond MF, Brien BJ, Torrance GW, Stoddart GL. *Methods for the Economic Evaluation of Health Care Programmes*.. 2nd ed. Oxford University Press; 1997.

31. *Methods for the development of NICE public health guidance*. [Online]. 3rd n. National Institute for Clinical Excellence (NICE); 2018. Available from: https://www.nice.org.uk/process/pmg4/resources/methods-for-the-development-of-nice-public-health-guidance-third-edition-pdf-2007967445701

32. Popay, J., Roberts, H., Sowden, A., Petticrew, M., Arai, L., Rodgers, M., ... & Duffy S. Guidance on the conduct of narrative synthesis in systematic reviews. A product from the ESRC methods programme. 2006;(April 2006): 1–92.

33. Duval S, Tweedie R. Trim and fill: A simple funnel-plot-based method of testing and adjusting for publication bias in meta-analysis. *Biometrics*. [Online] Biometric Society; 2000;56(2): 455–463. Available from: doi:10.1111/j.0006-341X.2000.00455.x [Accessed: 20th January 2021]

34. Guyatt GH, Oxman AD, Vist GE, Kunz R, Falck-Ytter Y, Alonso-Coello P, et al. GRADE: an emerging consensus on rating quality of evidence and strength of recommendations. *BMJ*. [Online] 2008;336(7650): 924–926. Available from: doi:10.1136/bmj.39489.470347.AD

35. Warren LR, Harrison M, Arora S, Darzi A. Working with patients and the public to design an electronic health record interface: A qualitative mixed-methods study. *BMC Medical Informatics and Decision Making*. [Online] BMC Medical Informatics and Decision Making; 2019;19(1): 1–8. Available from: doi:10.1186/s12911-019-0993-7

For peer review only

For peer review only - http://bmjopen.bmj.com/site/about/guidelines.xhtml

Authors' contributions

Conception and design of the work: EL, ALN, and JC wrote the manuscript. HA, and AD provided critical revision of drafts for important intellectual content. All authors provided input into drafts of the manuscript and agree on the contents of the final version.

Funding statement

This research was supported through the Imperial College National Institute for Health Research (NIHR) Patient Safety Translational Research Centre (PSTRC) and the Imperial College Biomedical Research Centre (BRC). JC acknowledges support from the Wellcome Trust [215938/Z/19/Z]. However, the funder/sponsor has had no role in development and drafting of this protocol.

Competing Interests

The authors declare that there are no competing interests.

Word count: 3886

For peer review only

Supplement 1: Search strategy exploring main themes, utilizing search terms and related terminology derivations for each theme (electronic health records, interoperability, and patient safety).

#	Searches
1	exp Medical Records Systems, Computerized/ or exp Electronic Health Records/ or exp Hospital Information Systems/
2	limit 1 to (english language and yr="2010 - 2020")
3	exp Electronic Health Records/
4	limit 3 to (english language and yr="2010 - 2020")
5	exp Health Information Exchange/ or exp Medical Informatics/ or exp Decision Support Systems, Clinical/ or exp Medical Records Systems, Computerized/ or exp Medical Record Linkage/
6	limit 5 to (english language and yr="2010 - 2020")
7	exp Medical Informatics/
8	limit 7 to (english language and yr="2010 - 2020")
9	exp Hospital Information Systems/
10	limit 9 to (english language and yr="2010 - 2020")
11	exp Medical Informatics/
12	limit 11 to (english language and yr="2010 - 2020")
13	exp Health Information Interoperability/
14	limit 13 to (english language and yr="2010 - 2020")
15	exp Systems Integration/
16	limit 15 to (english language and yr="2010 - 2020")
17	exp Patient Safety/
18	limit 17 to (english language and yr="2010 - 2020")
19	(Patient adj1 incident*).mp. [mp=title, abstract, original title, name of substance word, subject heading word, floating sub-heading word, keyword heading word, organism supplementary concept word, protocol supplementary concept word, rare disease supplementary concept word, unique identifier, synonyms]
20	limit 19 to (english language and yr="2010 - 2020")
21	(Adverse adj1 event*).mp. [mp=title, abstract, original title, name of substance word, subject heading word, floating sub-heading word, keyword heading word, organism supplementary

	concept word, protocol supplementary concept word, rare disease supplementary concept word, unique identifier, synonyms]
22	limit 21 to (english language and yr="2010 - 2020")
23	(Patient adj1 outcome*).mp. [mp=title, abstract, original title, name of substance word, subject heading word, floating sub-heading word, keyword heading word, organism supplementary concept word, protocol supplementary concept word, rare disease supplementary concept word, unique identifier, synonyms]
24	limit 23 to (english language and yr="2010 - 2020")
25	(Patient adj1 harm).mp. [mp=title, abstract, original title, name of substance word, subject heading word, floating sub-heading word, keyword heading word, organism supplementary concept word, protocol supplementary concept word, rare disease supplementary concept word, unique identifier, synonyms]
26	limit 25 to (english language and yr="2010 - 2020")
27	exp Risk Management/
28	limit 27 to (english language and yr="2010 - 2020")
29	2 or 4 or 6 or 8 or 10 or 12
30	14 or 16
31	18 or 20 or 22 or 24 or 26 or 28
32	29 and 30 and 31

For peer review only - http://bmjopen.bmj.com/site/about/guidelines.xhtml

PRISMA-P (Preferred Reporting Items for Systematic review and Meta-Analysis Protocols) 2015 checklist: recommended items to address in a systematic review protocol*

Section and topic	Item No.	Checklist item	Reported on Page No.
**ADMINISTRATIVE INFORMATION**			
Title:			
Identification	1a	Identify the report as a protocol of a systematic review	1
Update	1b	If the protocol is for an update of a previous systematic review, identify as such	N/A
Registration	2	If registered, provide the name of the registry (such as PROSPERO) and registration number	3
Authors:			
Contact	3a	Provide name, institutional affiliation, e-mail address of all protocol authors; provide physical mailing address of corresponding author	1
Contributions	3b	Describe contributions of protocol authors and identify the guarantor of the review	1
Amendments	4	If the protocol represents an amendment of a previously completed or published protocol, identify as such and list changes; otherwise, state plan for documenting important protocol amendments	N/A
Support:			
Sources	5a	Indicate sources of financial or other support for the review	8
Sponsor	5b	Provide name for the review funder and/or sponsor	8
Role of sponsor or funder	5c	Describe roles of funder(s), sponsor(s), and/or institution(s), if any, in developing the protocol	8
**INTRODUCTION**			
Rationale	6	Describe the rationale for the review in the context of what is already known	4-5
Objectives	7	Provide an explicit statement of the question(s) the review will address with reference to participants, interventions, comparators, and outcomes (PICO)	5
**METHODS**			
Eligibility criteria	8	Specify the study characteristics (such as PICO, study design, setting, time frame) and report characteristics (such as years considered, language, publication status) to be used as criteria for eligibility for the review	5-6
Information sources	9	Describe all intended information sources (such as electronic databases, contact with study authors, trial registers or other grey literature sources) with planned dates of coverage	5
Search strategy	10	Present draft of search strategy to be used for at least one electronic database, including planned limits, such that it could be repeated	5

For peer review only

Study records:			
Data management	11a	Describe the mechanism(s) that will be used to manage records and data throughout the review	6-7
Selection process	11b	State the process that will be used for selecting studies (such as two independent reviewers) through each phase of the review (that is, screening, eligibility and inclusion in meta-analysis)	6
Data collection process	11c	Describe planned method of extracting data from reports (such as piloting forms, done independently, in duplicate), any processes for obtaining and confirming data from investigators	6-7
Data items	12	List and define all variables for which data will be sought (such as PICO items, funding sources), any pre-planned data assumptions and simplifications	6
Outcomes and prioritization	13	List and define all outcomes for which data will be sought, including prioritization of main and additional outcomes, with rationale	6
Risk of bias in individual studies	14	Describe anticipated methods for assessing risk of bias of individual studies, including whether this will be done at the outcome or study level, or both; state how this information will be used in data synthesis	6-7
Data synthesis	15a	Describe criteria under which study data will be quantitatively synthesised	6-7
	15b	If data are appropriate for quantitative synthesis, describe planned summary measures, methods of handling data and methods of combining data from studies, including any planned exploration of consistency (such as I^2, Kendall's τ)	7
	15c	Describe any proposed additional analyses (such as sensitivity or subgroup analyses, meta-regression)	-
	15d	If quantitative synthesis is not appropriate, describe the type of summary planned	7
Meta-bias(es)	16	Specify any planned assessment of meta-bias(es) (such as publication bias across studies, selective reporting within studies)	6-7
Confidence in cumulative evidence	17	Describe how the strength of the body of evidence will be assessed (such as GRADE)	7

*** It is strongly recommended that this checklist be read in conjunction with the PRISMA-P Explanation and Elaboration (cite when available) for important clarification on the items. Amendments to a review protocol should be tracked and dated. The copyright for PRISMA-P (including checklist) is held by the PRISMA-P Group and is distributed under a Creative Commons Attribution Licence 4.0.**

From: Shamseer L, Moher D, Clarke M, Ghersi D, Liberati A, Petticrew M, Shekelle P, Stewart L, PRISMA-P Group. Preferred reporting items for systematic review and meta-analysis protocols (PRISMA-P) 2015: elaboration and explanation. BMJ. 2015 Jan 2;349(jan02 1):g7647.

For peer review only
